# N6-methyladenosine modified TGFB2 triggers lipid metabolism reprogramming to confer pancreatic ductal adenocarcinoma gemcitabine resistance

**DOI:** 10.1038/s41388-024-03092-3

**Published:** 2024-06-24

**Authors:** Ming-Jian Ma, Yin-Hao Shi, Zhi-De Liu, Ying-Qin Zhu, Guang-Yin Zhao, Jing-Yuan Ye, Fu-Xi Li, Xi-Tai Huang, Xi-Yu Wang, Jie-Qin Wang, Qiong-Cong Xu, Xiao-Yu Yin

**Affiliations:** 1 Department of Pancreato-Biliary Surgery, The First Affiliated Hospital of Sun Yat-sen University, Guangzhou, 510080 China; 2 Department of Animal Experiment Center, The First Affiliated Hospital of Sun Yat-sen University, Guangzhou, 510080 China; 3 Department of Key Laboratory of Stem Cells and Tissue Engineering, Sun Yat-sen University, Ministry of Education, Guangzhou, 510080 China; 4 Department of Pediatric Surgery, Guangzhou Women and Children’s Medical Center, Guangzhou Medical University, Guangzhou, 510623 China

**Keywords:** Chemotherapy, Oncogenes

## Abstract

Gemcitabine resistance is a major obstacle to the effectiveness of chemotherapy in pancreatic ductal adenocarcinoma (PDAC). Therefore, new strategies are needed to sensitize cancer cells to gemcitabine. Here, we constructed gemcitabine-resistant PDAC cells and analyzed them with RNA-sequence. Employing an integrated approach involving bioinformatic analyses from multiple databases, TGFB2 is identified as a crucial gene in gemcitabine-resistant PDAC and is significantly associated with poor gemcitabine therapeutic response. The patient-derived xenograft (PDX) model further substantiates the gradual upregulation of TGFB2 expression during gemcitabine-induced resistance. Silencing TGFB2 expression can enhance the chemosensitivity of gemcitabine against PDAC. Mechanistically, TGFB2, post-transcriptionally stabilized by METTL14-mediated m6A modification, can promote lipid accumulation and the enhanced triglyceride accumulation drives gemcitabine resistance by lipidomic profiling. TGFB2 upregulates the lipogenesis regulator sterol regulatory element binding factor 1 (SREBF1) and its downstream lipogenic enzymes via PI3K-AKT signaling. Moreover, SREBF1 is responsible for TGFB2-mediated lipogenesis to promote gemcitabine resistance in PDAC. Importantly, TGFB2 inhibitor imperatorin combined with gemcitabine shows synergistic effects in gemcitabine-resistant PDAC PDX model. This study sheds new light on an avenue to mitigate PDAC gemcitabine resistance by targeting TGFB2 and lipid metabolism and develops the potential of imperatorin as a promising chemosensitizer in clinical translation.

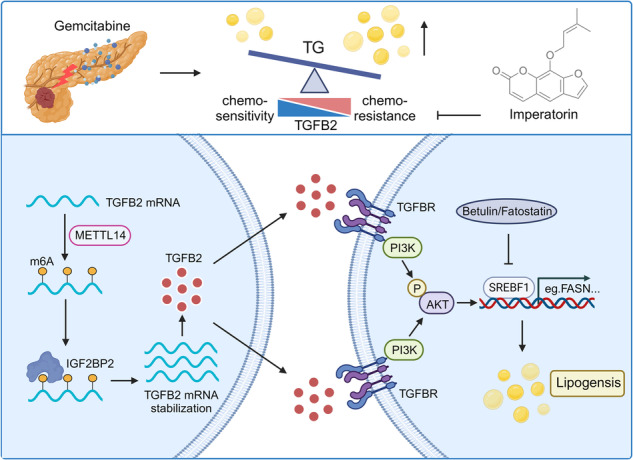

## Introduction

Pancreatic ductal adenocarcinoma (PDAC) is one of the most aggressive cancers with a dismal five-year survival rate of 10% [1], and almost 80% of patients present with an unresectable disease that has local or distant metastasis [2]. Gemcitabine-based combination chemotherapy strategies remain the dominant treatment for advanced PDAC [3, 4]. Initial sensitivity to gemcitabine therapy is frequently seen in PDAC patients, nevertheless, the rapid development of drug resistance severely limits the efficacy of this treatment regimen [5]. Thus, it is of fundamental importance to explore methods for improving the sensitivity of PDAC to gemcitabine and uncover new targets of gemcitabine resistance for the treatment of PDAC.

Gemcitabine resistance is caused by a number of intricate and varied factors, including aberrant DNA damage and repair, atypical signaling pathways, abnormal gemcitabine metabolism, etc. [6]. Accumulating evidence has revealed that gemcitabine resistance in PDAC is closely connected to metabolic reprogramming [7]. Increasing glycolytic flux and de novo pyrimidine biosynthesis led to gemcitabine resistance in pancreatic cancer cells [8, 9]; Gln addiction also fueled hexosamine biosynthesis and activated AKT pathway, contributing to chemoresistance [10, 11]. Besides the well-characterized reprogramming of glucose and amino acid metabolism, lipid metabolic reprogramming has emerged as an established trait of cancer metabolism during therapy response and desensitization [12]. The development of a resistant phenotype in tumor cells subjected to chemotherapy is sustained by enhanced lipogenesis, increased lipid content, and lipid-dependent catabolism [13]. However, the lipid metabolism underpinning gemcitabine resistance in PDAC has not yet been fully elucidated.

Transforming growth factor-beta (TGF-beta) superfamily includes TGF-beta itself, bone morphogenetic protein (BMP), and growth and differentiation factor (GDF), whose signaling dysfunction has been linked to cancer [14, 15]. Through tumor proliferation, tumor metastasis, tumor angiogenesis, and immune escape of tumor cells, TGF-beta can aid in the development of tumors [16–18]. It has been reported that high expression of TGFB2 (transforming growth factor-beta 2) in pancreatic cancer is substantially correlated with advanced tumor progression, and high serum levels of TGFB2 in patients are associated with shorter survival [19, 20]. Higher TGFB2 expression is associated with larger tumors, faster metastasis, and wider invasion [21]. Moreover, targeting TGF-beta signaling can inhibit the development of pancreatic cancer [22]. Recent studies have revealed that TGFB2 plays an essential role in the therapeutic response of malignancies [23–26]. For instance, TGFB2 induced epithelial-mesenchymal transition and activated the NF-κB pathway to contribute to osimertinib resistance in epidermal growth factor receptor (EGFR) mutant non-small cell lung cancer [27]. Our current bioinformatics analysis reveals that TGFB2 is associated with the lipid metabolic pathway in cancer drug sensitivity databases. Thus, we hypothesized that TGFB2 may act as a regulator of lipid metabolism to confer PDAC gemcitabine resistance.

In this study, we demonstrate that TGFB2 expression is abnormally elevated in gemcitabine-resistant PDAC cells, and patient-derived xenograft (PDX) models, even clinical tumor tissue. We show that silencing TGFB2 expression can significantly enhance the therapeutic effect of gemcitabine on PDAC both in vitro and in vivo. Mechanistically, TGFB2 expression is regulated by m6A modification mediated by METTL14 and dependent on the “reader” protein IGF2BP2. Aberrantly high-expressed TGFB2 promotes neutral lipids accumulation in gemcitabine-resistant PDAC. Specifically, the lipidomic analysis suggested that increased triglyceride accumulation drives gemcitabine resistance. We further elucidate that TGFB2 modulates gemcitabine sensitivity by AKT-SREBF1 signaling mediated lipid metabolism reprogramming. Furthermore, the combination strategy of TGFB2 inhibitor imperatorin and gemcitabine showed synergistic activities in the gemcitabine-resistant PDAC PDX model.

## Results

### TGFB2 is a crucial gene in gemcitabine-resistant PDAC and correlates with poor gemcitabine therapy response

**Fig. 1:**
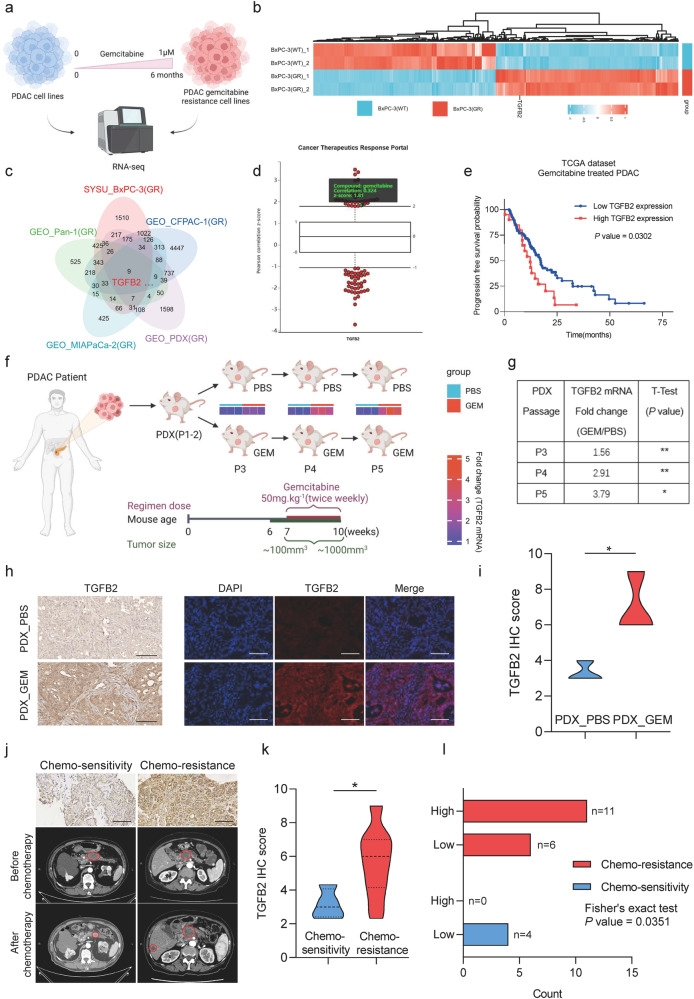
TGFB2 is a crucial gene in gemcitabine-resistant PDAC and correlates with poor gemcitabine therapy response. **a** Schematic diagram of gemcitabine-resistant cell construction of PDAC (Created with BioRender.com). **b** The heat plot showed differential genes in gemcitabine-resistant BxPC-3 cells (GR/WT). **c** Venn diagram showed intersecting differential genes in the drug resistance database. **d** High TGFB2 expression correlates with resistance to gemcitabine in PDAC cells. Plotted data were mined from the CTRP database. **e** Correlation between TGFB2 expression and progression-free survival in patients with PDAC treated with gemcitabine in TCGA dataset. **f** Schematic diagram of gemcitabine-resistant PDX construction of PDAC (Created with BioRender.com). Heatmap analysis of TGFB2 expression between PDX_GEM and PDX_PBS group. **g** The table shows the fold change and statistical analysis of TGFB2 mRNA level in gemcitabine-resistant PDX. **h** Representative images of IHC staining for TGFB2 in PDX tumor (scale bars = 100 μm). **i** Statistical analysis of IHC staining in PDX tumor. **j** Representative images of IHC staining for TGFB2 and CT in gemcitabine-resistant and gemcitabine-sensitive PDAC (scale bars = 100 μm). **k** Statistical analysis of IHC staining in PDAC tumor tissue. The data are shown as mean ± SD. *p < 0.05; ** *p* < 0.01; ***p < 0.001 according to Student’s *t*-test. **l** The correlation between high/low TGFB2 level and gemcitabine response was analyzed using Fisher’s exact test.

To identify genes associated with gemcitabine resistance, gemcitabine-resistant PDAC cell lines were established by subjecting BxPC-3/CFPAC-1 cells to escalating doses of gemcitabine (Fig. 1a). The half maximal inhibitory concentration (IC50) was distinctly higher in BxPC-3/CFPAC-1(GR) cells (IC50:749.8 nM; 743.8 nM) compared to wild type (WT) (IC50:3.30 nM; 1.7 nM) after gemcitabine induction for 6 months (Fig. S1(a)). Then we performed RNA sequencing on BxPC-3(GR) and BxPC-3(WT) cells (Fig. 1a, b), and combined multiple PDAC databases for bioinformatic analysis. The Venn diagram illustrated that TGFB2 expression was upregulated in gemcitabine-resistant cells (Fig. 1c). This was also confirmed by RT-qPCR in BxPC-3(WT/GR) cell and CFPAC-1(WT/GR) cell (Fig. S1(b)). We also demonstrated that gemcitabine induction increased the expression of TGFB2 both dose-dependently and time-dependently in BxPC-3 and CFPAC-1 cells (Fig. S1(c, d)). Analyses of the Cancer Therapeutics Response Portal (CTRP) revealed that TGFB2 expression correlated with resistance to gemcitabine in PDAC (Fig. 1d). Moreover, KM plotter analysis showed that high TGFB2 expression was associated with worse progression-free survival in patients treated with gemcitabine (Fig. 1e).

To further validate the result from bioanalysis, we successfully established PDX models from PDAC tissues and passed them to the second generation (P2). Then we passed the PDX (P3) tumors in BALB/C nude mice with the treatment of gemcitabine to P5. As shown in Fig. 1f, g, the TGFB2 mRNA level was significantly higher in PDX_GEM than PDX_PBS and the fold change increased with gemcitabine-induced generations. Similarly, IHC staining revealed that TGFB2 expression was elevated in PDX_GEM compared to PDX_PBS tumor tissue (Fig. 1h, i).

Next, TGFB2 expression levels in 17 chemotherapy-resistant and 4 chemotherapy-sensitive tissue specimens from our center were also examined to clarify the role of TGFB2 in PDAC gemcitabine chemotherapy (Fig. 1j), showing that TGFB2 was highly expressed in chemotherapy-resistant tissues (Fig. 1k). Patients were divided into high and low TGFB2 expression groups according to the relative expression level of TGFB2. In the group with a higher level of TGFB2, all patients were chemotherapy-resistant, while no patient showed chemotherapy-sensitivity (chemotherapy-resistance: 11 vs. 6; chemotherapy-sensitivity: 0 vs 4; *p* = 0.0351) compared with the group with a lower level of TGFB2 (Fig. 1l). Overall, these results suggested that TGFB2 overexpression plays a crucial role in gemcitabine-resistant PDAC and correlates with poor gemcitabine therapy response.

### TGFB2 expression is upregulated by N6‐methyladenosin (m6A) mediated mRNA stabilization

**Fig. 2:**
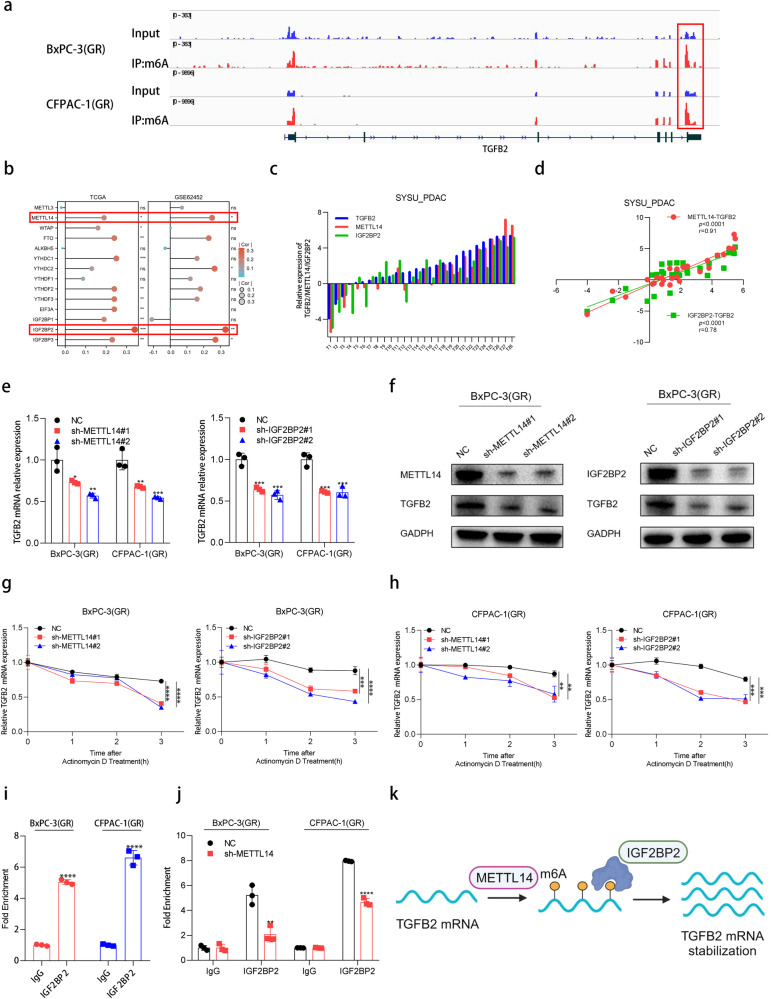
TGFB2 expression is upregulated by N6‐methyladenosin (m6A) mediated mRNA stabilization. **a** Integrative genomics viewer (IGV) plots indicate m6A peaks at TGFB2 mRNAs in MeRIP-seq of gemcitabine-resistant BxPC-3/CFPAC-1 cells. **b** Correlation between TGFB2 and m6A-related genes in TCGA and GEO databases. **c**, **d** RT-qPCR and correlation analysis revealed an evident correlation between TGFB2 and METTL14, IGF2BP2 mRNA expression in 28 PDAC tissues. **e** The mRNA level of TGFB2 after METTL14 or IGF2BP2 knockdown in BxPC-3(GR) and CFPAC-1(GR) cells was confirmed by RT-qPCR. **f** The protein level of TGFB2 after METTL14 or IGF2BP2 knockdown in BxPC-3(GR) cells was confirmed by Western blotting. **g** RT-qPCR analysis of TGFB2 after actinomycin D treatment in sh-METTL14 or sh-IGF2BP2 BxPC-3(GR) cells. **h** RT-qPCR analysis of TGFB2 after actinomycin D treatment in sh-METTL14 or sh-IGF2BP2 CFPAC-1(GR) cells. **i** RNA immunoprecipitation with an anti-IGF2BP2 antibody was used to assess whether IGF2BP2 binding to TGFB2 in PDAC(GR) cells, IgG was used as the control. **j** RIP-qPCR by using anti-IGF2BP2 antibody showed the affinity of TGFB2 mRNA to IGF2BP2 in sh-METTL14 cells. The data are shown as mean ± SD. *p < 0.05; **p < 0.01; ***p < 0.001 according to Student’s *t*-test. **k** Illustration of the mechanism by which TGFB2 is modified by METTL14‐mediated m6A, followed by mRNA stabilization via IGF2BP2‐dependent recognition (Created with BioRender.com).

The most prominent internal RNA modification, N6-methyladenosine (m6A), has been involved in the regulation of RNA stability, splicing, and degradation [28, 29]. To elucidate the reasons for the abnormally high expression of TGFB2 in gemcitabine-resistant PDAC, methylated RNA immunoprecipitation-sequence (MeRIP-seq) was performed in PDAC(GR) cells. The m6A peak in TGFB2 3’UTR is extremely prominent in MeRIP-seq of both BxPC-3(GR) and CFPAC-1(GR) cells (Fig. 2a). We further analyzed the relationship between m6A-related genes and TGFB2, suggesting that TGFB2 significantly related to METTL14 and IGF2BP2 in both TCGA and GEO database (Fig. 2b). Consistently, TGFB2 expression was positively associated with METTL14 and IGF2BP2 mRNA expression in PDAC tissue samples from our PDAC cohort (Fig. 2c, d). We then knocked down METTL14 and IGF2BP2 mRNA using specific shRNA, respectively, (Fig. S2(a)) and found that knockdown of METTL14 and IGF2BP2 significantly reduced TGFB2 mRNA levels in BxPC-3(GR) and CFPAC-1(GR) cells (Fig. 2e). Meanwhile, the protein level of TGFB2 was also reduced (Fig. 2f and Fig. S2(b)). Additionally, MeRIP-qPCR indicated that the m6A modification was significantly enriched in TGFB2 mRNA, but decreased in METTL14-knockdown BxPC-3(GR) and CFPAC-1(GR) cells (Fig. S2(c)). Next, actinomycin D assay showed that the knockdown of METTL14 and IGF2BP2 could decelerate the mRNA stability of TGFB2 (Fig. 2g, h). Moreover, the RIP assay revealed the enrichment of TGFB2 in anti-IGF2BP2 immuno-precipitates compared with the IgG control (Fig. 2i). And a notably reduced affinity of IGF2BP2 to TGFB2 in METTL14-silenced BxPC-3(GR) and CFPAC-1(GR) cells was observed (Fig. 2j and Fig. S2(d)). Overall, our results revealed that TGFB2 is modified by METTL14‐mediated m6A, followed by mRNA stabilization via IGF2BP2‐dependent recognition.

### TGFB2 knockdown potentiates PDAC gemcitabine efficacy in vitro and in vivo

**Fig. 3:**
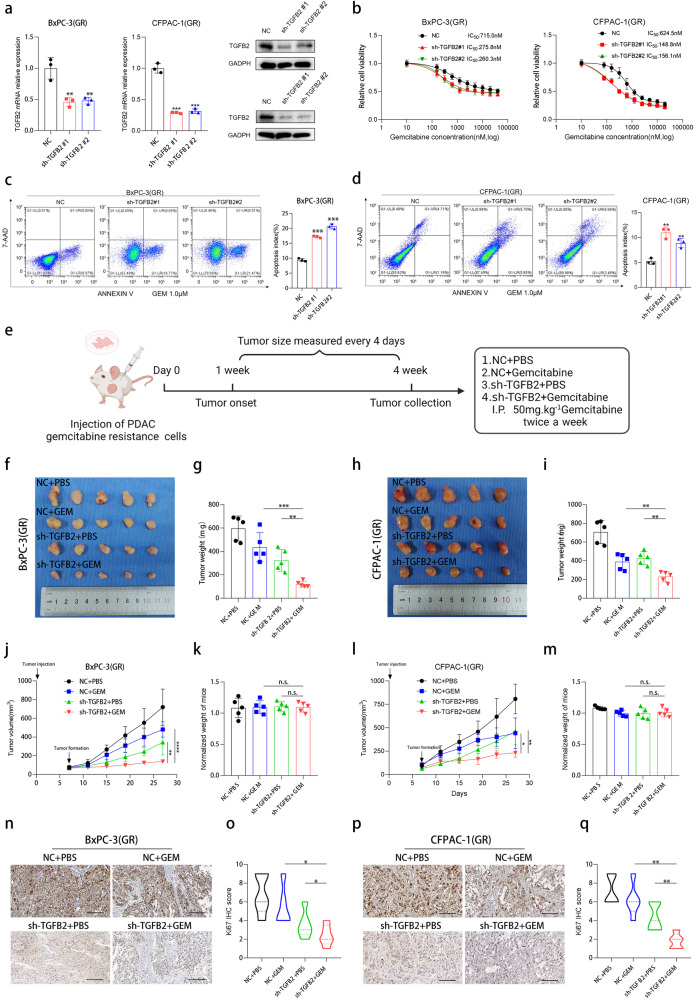
TGFB2 knockdown potentiates PDAC gemcitabine efficacy in vitro and in vivo. **a** TGFB2 expression after TGFB2 knockdown in BxPC-3(GR) and CFPAC-1(GR) cells was confirmed by RT-qPCR and western blot. **b** The IC50 value changes after knocking down TGFB2 in BxPC-3(GR) and CFPAC-1(GR) cells. **c**, **d** Apoptosis assay treated with 1 μM gemcitabine in BxPC-3(GR) and CFPAC-1(GR) cells. **e** Schematic diagram of xenografts in BALB/c nude mice by inoculating PDAC(GR) cells at their right armpits (Created with BioRender.com). **f** Xenograft tumors derived from BxPC-3(GR) cells were shown. **g** Tumor weight of each BxPC-3(GR) group. **h** Xenograft tumors derived from CFPAC-1 (GR) cells were shown. **i** Tumor weight of each CFPAC-1(GR) group. **j** Tumor growth curves after the injection of sh-TGFB2 and NC of BxPC-3(GR) cells. **k** The final weight of mice in each BxPC-3(GR) group randomized to the initial weight. **l** Tumor growth curves after the injection of sh-TGFB2 and NC of CFPAC-1(GR) cells. **m** The final weight of mice in each CFPAC-1(GR) group randomized to the initial weight. **n** Representative IHC staining of Ki67 in tumors from each BxPC-3(GR) group (scale bars = 100 μm). **o** Statistical analysis of IHC staining of Ki67 in tumors from each BxPC-3(GR) group. **p** Representative IHC staining of Ki67 in tumors from each CFPAC-1(GR) group (scale bars = 100 μm). **q** Statistical analysis of IHC staining of Ki67 in tumors from each CFPAC-1(GR) group. The data are shown as mean ± SD. *p < 0.05; **p < 0.01; ***p < 0.001 according to Student’s *t*-test.

To investigate the potential role of TGFB2 in gemcitabine resistance in PDAC, TGFB2 expression was suppressed by shRNA transfection in BxPC-3(GR) and CFPAC-1(GR) cells (Fig. 3a). To determine the effect of TGFB2 on gemcitabine sensitivity, we next treated the cells with complete medium with indicated concentration gradient of gemcitabine for 72 h, finding that the half maximal inhibitory concentration (IC50) dramatically decreased in the sh-TGFB2 group compared with the NC group (Fig. 3b). Then we examined the effect of TGFB2 on the cell proliferative ability under gemcitabine treatment. Downregulation of TGFB2 substantially reduced the rates of cell proliferation of PDAC gemcitabine-resistant cells (Fig. S3(a)). Furthermore, we treated the shTGFB2 and NC cells with indicated gemcitabine concentration for 72 h for apoptosis detection. The results showed that silencing the expression of TGFB2 significantly increased cell apoptosis (Fig. 3c, d). Additionally, recombinant human TGFB2 protein was also used to simulate PDAC gemcitabine-resistant cells. As anticipated, overexpression of TGFB2 strengthened the gemcitabine resistance of PDAC cells assessed by IC50 tests (Fig. S3(b)). Apoptosis assay showed that overexpression of TGFB2 reduced cell apoptosis (Fig. S3(c)).

To assess whether the expression level of TGFB2 could affect gemcitabine-resistant PDAC cell growth in vivo, TGFB2 stable knockdown PDAC gemcitabine-resistant cells and their control cells were subcutaneously injected into BALB/c nude mice (Fig. 3e). We found that the TGFB2-deficient tumors were more vulnerable to gemcitabine than NC group. The tumor volume and weight were significantly lower in the shTGFB2-transfected with gemcitabine treatment group (Fig. 3f–j, l). Interestingly, there was no significant difference in body weight among the four groups (Fig. 3k, m). Moreover, the proliferation marker Ki67 and PCNA were dramatically downregulated in the TGFB2 knockdown with gemcitabine treatment tumors (Fig. 3n–q and Fig. S3(d–g)). These findings indicated that the downregulation of TGFB2 could reshape the sensitivity of PDAC to gemcitabine.

### TGFB2 promotes neutral lipid accumulation by upregulating the expression of lipid synthetase in gemcitabine-resistant PDAC

**Fig. 4:**
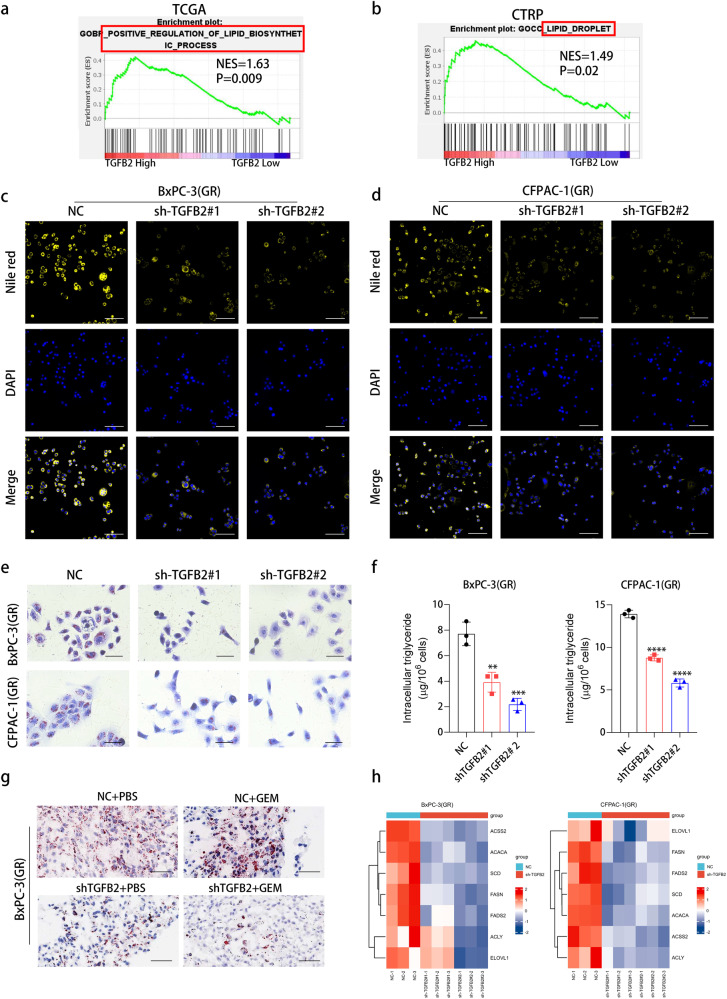
TGFB2 promotes neutral lipid accumulation by upregulating the expression of lipid synthetase in gemcitabine-resistant PDAC. **a**, **b** GSEA enrichment between high and low TGFB2 expression group from TCGA and CTRP database. **c** Cellular neutral lipids were measured in BxPC-3(GR) cells expressing NC, sh-TGFB2#1, and sh-TGFB2#2 by Nile red staining (scale bars = 100 μm). **d** Cellular neutral lipids were measured in CFPAC-1(GR) cells expressing NC, sh-TGFB2#1, and sh-TGFB2#2 by Nile red staining (scale bars = 100 μm). **e** Cellular neutral lipids were measured in BxPC-3(GR) and CFPAC-1(GR) cells expressing NC, sh-TGFB2#1, and sh-TGFB2#2 by Oil red O staining (scale bars = 50 μm). **f** Cellular triglyceride content was measured in BxPC-3(GR) and CFPAC-1(GR)cells expressing NC, sh-TGFB2#1 and sh-TGFB2#2. The values were normalized to cell counting. Data are presented as mean ± SD. *p < 0.05; **p < 0.01; ***p < 0.001 according to Student’s *t*-test. **g** Tissue-neutral lipids were measured in xenograft tumors with BxPC-3(GR) cells by Oil red O staining (scale bars = 50 μm). **h** Heat plot showed expression change in genes related to lipid synthesis in BxPC-3(GR) and CFPAC-1(GR)cells expressing NC, sh-TGFB2#1, and sh-TGFB2#2.

To investigate mechanisms as to how TGFB2 regulates gemcitabine resistance in PDAC, we first divided patients into high and low TGFB2 expression groups according to the median value of TGFB2 expression in the TCGA PDAC database and found that TGFB2 could positively regulate lipid biosynthesis using Gene set enrichment analysis (GSEA) (Fig. 4a). Similarly, by submitting Cancer Therapeutics Response Portal (CTRP) data we found TGFB2 was also involved in lipid metabolism, that is lipid droplet (Fig. 4b). As abnormal lipid metabolism has been suggested to be involved in tumor progression, we wondered whether TGFB2 could affect lipid metabolism in PDAC gemcitabine-resistant cells. Knock-down of TGFB2 in BxPC-3(GR) and CFPAC-1(GR) cells suppressed cellular lipid accumulation as shown by Nile red staining and Oil red O staining (Fig. 4c–e). Meanwhile, the level of intracellular triglyceride was significantly lower in TGFB2-silenced cells, compared to control cells (Fig. 4f). Furthermore, Oil red O staining of tumor tissue indicated that TGFB2 knockdown could decrease lipid accumulation (Fig. 4g and Fig. S4(a)). Inversely, treating gemcitabine-resistant cells with recombinant human TGFB2 protein promoted cellular lipids accumulation (Fig. S4b, c). Interestingly, the levels of the key lipogenic enzymes were significantly downregulated after TGFB2 knockdown in BxPC-3(GR) and CFPAC-1(GR) cells (Fig. 4h). Treating gemcitabine-resistant cells with recombinant human TGFB2 protein upregulated levels of genes related to lipid synthesis (Fig. S4(d, e)). Together, our findings suggest that inhibiting TGFB2 expression could downregulate the expression of lipid synthetase and reduce neutral lipid accumulation in gemcitabine-resistant PDAC.

**Fig. 5:**
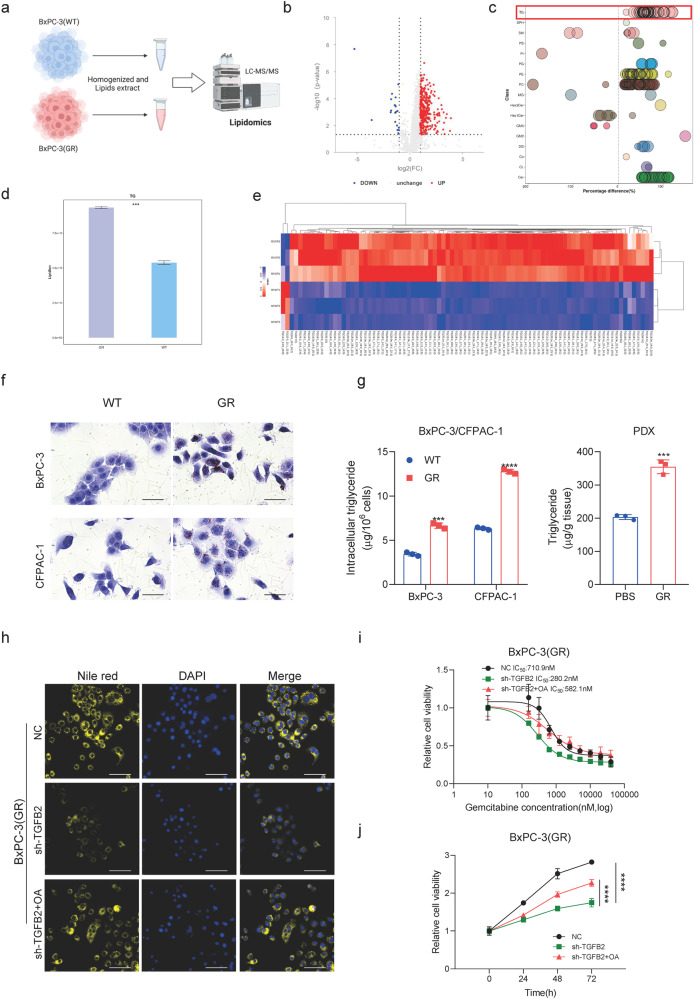
Increased triglyceride accumulation contributes to promoting gemcitabine resistance in PDAC. **a** Flow chart of lipidomic analysis in BxPC-3(WT) and BxPC-3(GR) cells (Created with BioRender.com). **b** Volcano plot showed differential lipid molecules of gemcitabine resistance. **c** Bubble plot showed the differential lipid subclasses and molecules of gemcitabine resistance. **d** Total content of triglyceride in BxPC-3(WT) and BxPC-3(GR) cells. **e** Heat plot showed detailed triglyceride content in BxPC-3(WT) and BxPC-3(GR) cells. **f** Cellular neutral lipids were measured in BxPC-3(WT/GR) and CFPAC-1(WT/GR) cells by Oil red O staining (scale bars = 50 μm). **g** Triglyceride content was measured in BxPC-3(WT/GR) cells, CFPAC-1(WT/GR) cells, and PDX(PBS/GR). **h** Cellular neutral lipids were measured in BxPC-3(GR) cells with treatment as indicated by Nile red staining (scale bars = 100 μm). **i** The IC50 value changes with treatment as indicated in BxPC-3(GR) cell. **j** Cell growth curve of BxPC-3(GR) cell with treatment as indicated.

How does lipid accumulation contribute to the drug resistance of PDAC? In recent years, mounting evidence has demonstrated that lipid metabolism is reprogrammed to adjust to changes in the tumor microenvironment and nutrient requirements during the occurrence, development, and metastasis of tumor cells [30, 31]. Abnormal lipid metabolism provides energy, biofilm, and signaling molecules for tumor cells, which affects the sensitivity and tolerance of tumor cells to chemotherapy drugs [13]. To further explore the role of lipid metabolism in PDAC gemcitabine resistance, a high-quality untargeted lipidomic analysis of BxPC-3(GR) and BxPC-3(WT) cells was performed (Fig. 5a). In general, 40 classes of lipids, which were composed of 360 triglycerides (TG), 357 phosphatidylcholines (PC), 232 phosphatidylethanolamine (PE), and other lipid classes, were screened and identified based on an LC-MS/MS system (Fig. S5(a)). Lipid molecules with significant abundance differences between BxPC-3(GR) and BxPC-3(WT) cells are depicted in a volcano plot in Fig. 5b. Among all the differentially altered lipids, triglyceride is one of the top upregulated lipid classes in BxPC-3(GR) cells compared with wild type (Fig. 5c and Fig. S5(b)). The chord diagram of the lipid-lipid correlation matrix suggested that triglyceride is the core metabolite related to most lipid metabolites (Fig. S5(c)). The gemcitabine-resistant cell has higher contents of triglyceride compared to the gemcitabine-sensitive cell (Fig. 5d). All the systematic lipidomic changes belonging to triglyceride were shown in detail using a heat plot (Fig. 5e). Then we validated the increased accumulation of triglyceride in gemcitabine-resistant PDAC cells compared with their wild type, as analyzed using Nile red staining and Oil Red O staining (Fig. S5(e, f) and Fig. 5f). Additionally, we detected the severity of steatosis by Oil Red O staining and found elevated lipid contents in gemcitabine-resistant PDAC PDX tumors (Fig. S5(d)). Furthermore, levels of triglyceride increase in gemcitabine-resistant PDAC cells or PDX tumors compared to controls (Fig. 5g). These results suggest that increased triglyceride accumulation may play a pivotal role in promoting gemcitabine resistance in PDAC. To further determine whether lipids accumulation was responsible for the TGFB2-mediated gemcitabine resistance in PDAC cells, we conducted rescue experiments by the provision of oleic acid in TGFB2-knockdown cells. The results showed that TGFB2 knockdown significantly reduced the intensity of lipid drop staining and triglyceride level, as well as the gemcitabine IC50 and cell proliferation ability in PDAC gemcitabine-resistant cells, whereas provision of oleic acid significantly restored the reduction (Fig. 5h–j and Fig. S6). Taken together, we concluded that TGFB2 promotes neutral lipids accumulation to confer PDAC gemcitabine resistance.

### SREBF1 is critical for TGFB2-AKT-mediated lipid synthesis to promote gemcitabine resistance in PDAC

**Fig. 6:**
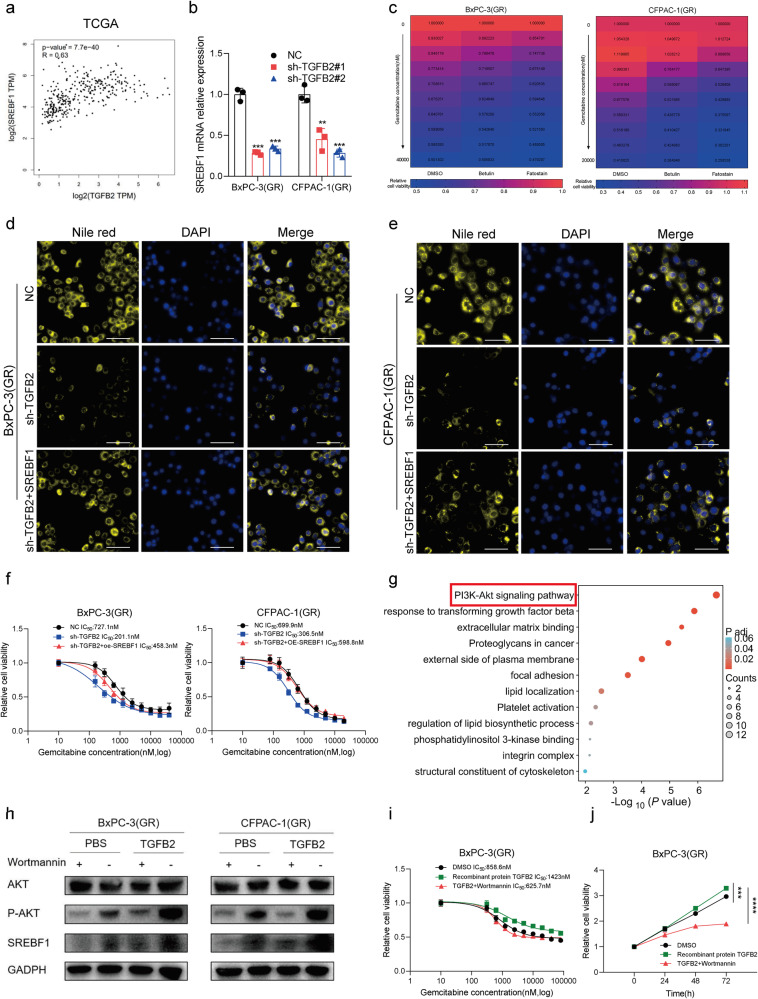
SREBF1 is critical for TGFB2-AKT mediated lipid synthesis to promote gemcitabine resistance in PDAC. **a** TGFB2 positively correlates with SREBF1 in the TCGA database. **b** The SREBF1 level after knockdown of TGFB2 in BxPC-3(GR) and CFPAC-1(GR) cells. **c** The gemcitabine IC50 value changes in BxPC-3(GR) and CFPAC-1(GR) cells treated with SREBF1 inhibitors. **d** Cellular neutral lipids were measured in BxPC-3(GR) cells with treatment as indicated by Nile red staining (scale bars = 100 μm). **e** Cellular neutral lipids were measured in CFPAC-1 (GR) cells with treatment as indicated by Nile red staining (scale bars = 100 μm). **f** The IC50 value changes in BxPC-3(GR) and CFPAC-1(GR) cells treated as indicated. **g** KEGG and GO analysis between high and low TGFB2 expression group from TCGA database. **h** The AKT, phosphorylated AKT (p-AKT), and SREBF1 protein levels after treatment with PI3K inhibitor wortmannin. **i** The IC50 value changes with treatment to recombinant protein TGFB2 and wortmannin as indicated in BxPC-3(GR) cells. **j** Cell growth curve of BxPC-3(GR) cell with treatment to recombinant protein TGFB2 and wortmannin as indicated.

It is well-known that SREBF1 is a key transcription factor for lipid synthesis [32]. Analysis of the TCGA dataset revealed a substantial positive association between TGFB2 and SREBF1 (Fig. 6a). The RT-qPCR analyses also showed that SREBF1 expression was significantly lower in both BxPC-3(GR) and CFPAC-1(GR) cells expressing TGFB2-sh1 or TGFB2-sh2 compared to NC cells (Fig. 6b). And treating gemcitabine-resistant cells with recombinant human TGFB2 protein could upregulate level of SREBF1 (Fig. S7(a)). Then we test the effect of SREBF1 on gemcitabine resistance. Betulin and fatostatin, the SREBF1 inhibitors, were used to treat gemcitabine-resistant cells. We found that they could reduce cell triglyceride (Fig. S7(b–d)) and strengthen the sensitivity of chemo-resistant cell lines to gemcitabine (Fig. 6c and Fig. S7(e)). To further determine whether the expression of SREBF1 was responsible for the TGFB2-mediated gemcitabine resistance in PDAC cells, we overexpressed SREBF1 in TGFB2-knockout cells (Fig. S7(f)). Our results showed that TGFB2 knockdown significantly reduced the cell triglyceride level and intensity of lipid drop staining, as well as the gemcitabine IC50 in PDAC gemcitabine-resistant cells, whereas overexpression of SREBF1 significantly restored the reduction (Fig. 6d–f and Fig. S7(g, h)).

Next, we wondered how TGFB2 facilitated the expression of SREBF1. Analysis of the TCGA and CTRP dataset found that the differentially expressed genes between the high and low TGFB2 expression groups were enriched in the PI3K/AKT signaling pathway, not only the lipid metabolism pathway (Fig. 6g and Fig. S8(a)). The PI3K-Akt signaling pathway has been linked to lipid metabolism in a number of studies and has been shown to activate the transcription factor SREBF1 [33, 34]. To validate the above findings, we evaluate the protein level of the key markers in the PI3K/AKT pathway and lipid synthesis process. After TGFB2 knockdown, the levels of PI3K, phosphorylated AKT and SREBF1 were significantly downregulated, while the protein levels of non-phosphorylated AKT were not significantly changed (Fig. S8(b)). To further study the involvement of PI3K/Akt, we treated gemcitabine-resistant cells with wortmannin, a PI3K inhibitor. Our results showed that wortmannin treatment markedly decreased the phosphorylation level of AKT in gemcitabine-resistant cells with or without TGFB2 overexpression. Meanwhile, the expression levels of SREBF1 were remarkably decreased by wortmannin treatment (Fig. 6h). Moreover, we treated TGFB2 overexpressed cells with wortmannin and assessed whether this treatment could reverse their phenotype. The results revealed that wortmannin remarkably suppressed the lipogenesis-promoting effect of TGFB2 overexpression (Fig. S8(c–e)). TGFB2 overexpression significantly strengthened the gemcitabine resistance of PDAC cells, which was significantly attenuated by treatment with wortmannin (Fig. 6i, j and Fig. S8(f, g)). Collectively, these data indicated that TGFB2 regulates gemcitabine sensitivity by AKT-SREBF1 signaling mediated lipid metabolism reprogramming.

### TGFB2 inhibitor imperatorin exhibits synergistic therapeutic effects with gemcitabine on PDAC

**Fig. 7:**
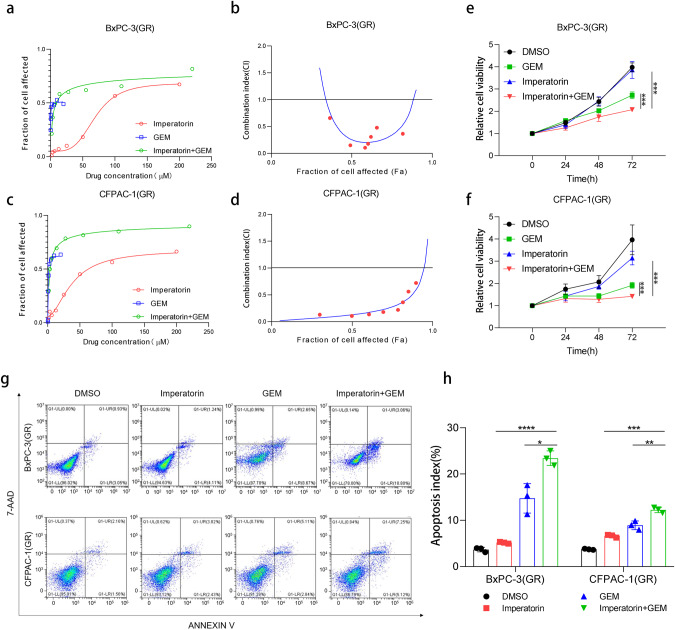
TGFB2 inhibitor imperatorin exhibits synergistic therapeutic effects with gemcitabine on PDAC. **a**, **b** The dose-effect curve and combination index of gemcitabine and imperatorin in BxPC-3(GR) cells. **c**, **d** The dose-effect curve and combination index of gemcitabine and imperatorin in CFPAC-1(GR) cells. **e** Cell viability assay of BxPC-3(GR) in different treatment groups. **f** Cell viability assay of CFPAC-1(GR) cells in different treatment groups. **g** The apoptosis assay with imperatorin and gemcitabine treatment in BxPC-3(GR) and CFPAC-1(GR) cells. **h** Statistical analysis of apoptosis assay with imperatorin and gemcitabine treatment in BxPC-3(GR) and CFPAC-1(GR) cells. Data are presented as mean ± SD. *p < 0.05; **p < 0.01; ***p < 0.001 according to Student’s *t*-test.

To further explore the clinical translational value of targeting TGFB2, we identified two TGFB2 inhibitors through a literature review [35, 36]. The IC50 test of PDAC gemcitabine-resistant cells showed that imperatorin had a higher rate of growth inhibition (Fig. S9(a, b)). It is reported that imperatorin was verified as a novel TGFB2 inhibitor that can inhibit TGFB2 expression to suppress esophageal cancer metastasis [36], which also inhibited TGFB2 expression in PDAC gemcitabine-resistant cells (Fig. S9(c)). It was also found that after imperatorin treatment, the levels of PI3K, phosphorylated AKT, and SREBF1 were downregulated with no significant change in non-phosphorylated AKT (Fig. S9(d)). Then we wondered whether imperatorin could enhance the anti-tumor effect of gemcitabine in PDAC. To address the question, we performed a drug combination assay in PDAC gemcitabine-resistant cells. The finding indicated that imperatorin could markedly decrease the gemcitabine IC50 value in BxPC-3(GR) and CFPAC-1(GR) cells and imperatorin plus gemcitabine regimen had a promising synergistic effect (Fig. 7a–d). To further evaluate the efficacy of the imperatorin plus gemcitabine regimen of drug-resistant PDAC cells, we next examined the anti-tumor effect of the regimen by cell proliferative ability and apoptotic assay. The results indicated that the imperatorin plus gemcitabine regimen could significantly inhibit the proliferative ability of gemcitabine-resistant cells compared with gemcitabine or imperatorin alone (Fig. 7e, f). Moreover, the cell apoptotic rates in the combination therapy group were dramatically increased compared with the two single-drug therapy groups, indicating that imperatorin could enhance the anti-tumor effect of gemcitabine (Fig. 7g, h). These findings showed that inhibition of TGFB2 by imperatorin could effectively sensitize PDAC gemcitabine-resistant cells in vitro.

### Imperatorin can significantly enhance the therapeutic efficacy of gemcitabine against chemo-resistant PDAC PDX

**Fig. 8:**
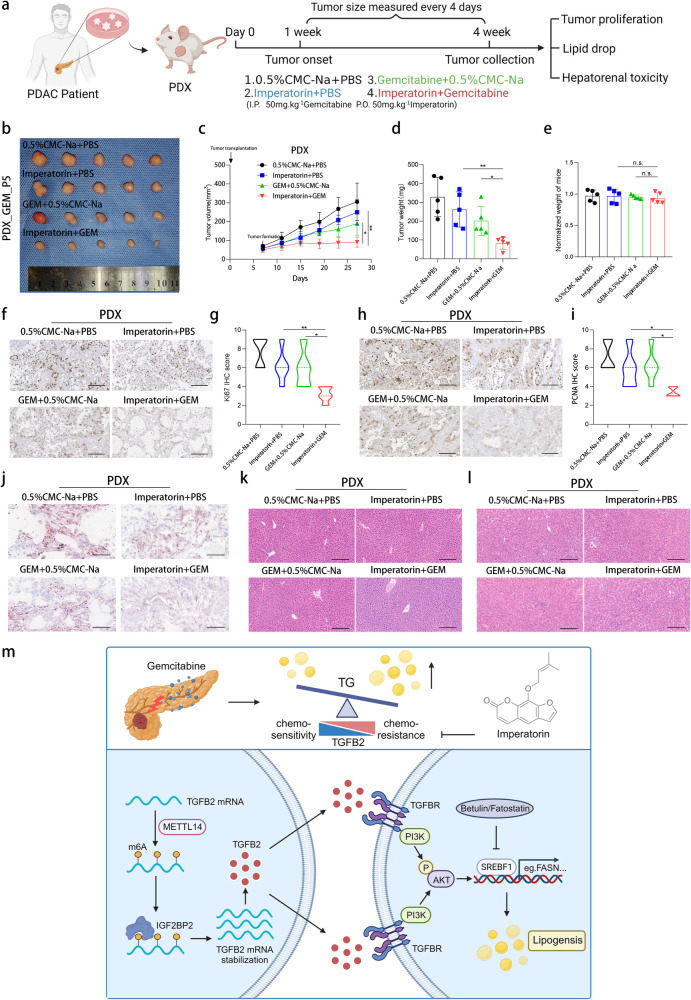
Imperatorin can significantly enhance the therapeutic efficacy of gemcitabine against chemo-resistant PDAC PDX. **a** Schematic diagram of PDX in BALB/c nude mice with imperatorin and gemcitabine treatment (Created with BioRender.com). **b** The tumors from 0.5%CMC-Na+PBS, Imperatorin+PBS, GEM + 0.5%CMC-Na, and Imperatorin+GEM group were shown. **c** Tumor growth curves of each group. **d** Tumor weight of each group. **e** The normalized weight of mice was shown. **f** Representative IHC staining of Ki67 in tumors with different treatments (scale bars = 100 μm). **g** Statistical analysis of IHC staining of Ki67 in tumors from different groups. **h** Representative IHC staining of PCNA in tumors with different treatments (scale bars = 100 μm). **i** Statistical analysis of IHC staining of PCNA in tumors from different groups. **j** Tissue-neutral lipids were measured in PDX tumors by Oil red O staining (scale bars = 50 μm). **k**, **l** HE staining (scale bars=200μm) of liver and kidney in the nude mice from different groups. The data are shown as mean ± SD. *p < 0.05; **p < 0.01; ***p < 0.001 according to Student’s *t-*test. **m** Schematic representation of the function and potential mechanism of TGFB2 in PDAC gemcitabine resistance (Created with BioRender.com).

We employed PDX_GEM P5 model to further investigate the therapeutic effect of the imperatorin plus gemcitabine regimen in vivo. We administered gemcitabine or the TGFB2 inhibitor imperatorin or a combination of both drugs to nude mice and continuously measured tumor size. The tumor growth in each group was observed every 4 days (Fig. 8a). We found that the imperatorin plus gemcitabine regimen significantly inhibited the proliferation of PDX tumors compared with the PBS group or each monotherapy group (Fig. 8b, c). The tumor weight at the end of the experiment was markedly decreased after imperatorin plus gemcitabine treatment, whereas the weight of mice in the four groups was not influenced by the combination treatment (Fig. 8d, e). In addition, IHC staining indicated the Ki67 and PCNA expression levels were significantly lower in the combination treatment group, which confirmed the powerful anti-tumor effect of imperatorin plus gemcitabine regimen in PDAC (Fig. 8f–i). The SREBF1 and p-AKT expression in the combination treatment group was also significantly lower than that either in the imperatorin or gemcitabine group (Fig. S10). Oil red O staining of PDX tumor tissue indicated that inhibition of TGFB2 could decrease lipid accumulation (Fig. 8j). Furthermore, HE staining of the liver and kidney showed that imperatorin had no significant toxic effect (Fig. 8k, l). These findings indicated that imperatorin had a potential effect on reversing PDAC gemcitabine resistance without a significant increase in side effects.

## Discussion

Chemoresistance has always been a major problem in PDAC treatment owing to the rapid progression of the disease [6]. Therefore, it is urgent to elucidate the underlying biological mechanisms and overcome gemcitabine resistance to improve chemosensitivity. In this study, we identified TGFB2 as a potential therapeutic target imparting gemcitabine resistance to PDAC. High-expressed TGFB2 in gemcitabine-resistant PDAC was post-transcriptionally stabilized by aberrant m6A modification. We also demonstrate that TGFB2 induces resistance to gemcitabine in PDAC both in vitro and in vivo and that the combination of TGFB2 inhibitor imperatorin and gemcitabine has a synergistic effect in PDAC PDX. Mechanistically, we confirmed that TGFB2 regulates gemcitabine sensitivity by PI3K/AKT signaling mediated SREBF1-dependent lipogenesis. Intriguingly, we find that increased triglyceride accumulation is a critical factor in acquiring resistance to gemcitabine in PDAC.

TGFB2 is a member of the TGF-beta superfamily, which has become one of the most widely researched growth factor families [37]. In addition to having an impact on the malignant development of several cancers, TGFB2 has recently been discovered to alter the chemotherapeutic response of cancers. For example, TGFB2 can mediate epithelial-mesenchymal transition and NF-κB pathway activation, thereby conferring resistance to osimertinib in non-small cell lung cancer [27]. In colorectal cancer, cancer-associated fibroblasts (CAFs)-secreted TGFB2 and hypoxia-inducible factor (HIF-1α) interact to activate the expression of hedgehog transcription factor GLI2 in cancer stem cells, resulting in intrinsic resistance to chemotherapy [25]. Two other studies in breast cancer also showed that inhibition of TGFB2 expression can maintain tumor sensitivity to chemotherapy [24, 26]. However, the role or molecular mechanism of TGFB2 in response to gemcitabine in PDAC remains unclear. We provided the first evidence suggesting TGFB2 as a key molecule promoting PDAC resistance by integrated bioinformatics analysis of multiple databases. Further studies showed that m6A mediated aberrantly overexpressed TGFB2 in PDAC could predict poor gemcitabine therapy response and TGFB2 knockdown potentiates PDAC gemcitabine efficacy in vitro and in vivo, thereby indicating TGFB2 as a candidate target to improve PDAC sensitivity to gemcitabine.

Gemcitabine has been coupled with a number of targeted treatments in clinical studies, such as EGFR, Smoothened, and vascular endothelial growth factor inhibitors. However, the results of these trials were mainly unfavorable, with no improvement in overall survival or progression-free survival [38]. It is imperative to seek other targeted treatments to efficiently improve chemosensitivity in PDAC. In this context, the response to the co-administration of TGFB2 inhibitor imperatorin and gemcitabine observed in the PDAC PDX is encouraging. It has been previously reported that imperatorin, a naturally occurring furanocoumarin, exerted a significantly inhibitory effect on TGFB2 expression [36] and it plays a pivotal role in tumor therapy in various cancers. In human larynx cancer, imperatorin was identified as a promising chemotherapeutic agent owing to inhibition of the cell cycle progression [39]. In human liver cancer, imperatorin acted as a chemosensitizer via downregulating Mcl-1 expression and cooperatively triggering Bax translocation and Bak activation [40, 41]. Similarly, we found that the combination of TGFB2 inhibitor imperatorin with gemcitabine significantly improved tumor response in gemcitabine-resistant PDAC PDX. This is in accordance with the synergistic effects of the two drugs that we observed in vitro. The mice weight monitoring and hepatorenal HE staining provide evidence of a low degree of toxicity for imperatorin, raising the possibility that a combination of imperatorin and gemcitabine can be translated into the clinical setting to treat PDAC.

Reprogrammed lipid metabolism has been recognized as a common metabolic hallmark in many cancers. Increased lipid synthesis supplies cancer cells with energy storage, building blocks, and signaling molecules to promote rapid cancer cell growth and tumor formation. It is reported that TGFB2 was involved in fatty acid metabolism [42] and TGFB2 signaling favored the formation of lipid droplets to support acidosis-driven EMT and the metastatic spreading of cancer cells [43]. In our study, the TCGA and CTRP database analysis revealed that high TGFB2-expressing PDAC tumors or cells relied significantly on lipid metabolism. From the PDAC gemcitabine-resistant cells and subcutaneous tumor, the detection of lipid droplets and their components further suggested that silencing TGFB2 reduced neutral lipid accumulation in gemcitabine-resistant PDAC. Combined with the fact that TGFB2 knockdown can mitigate chemoresistance (Fig. 3), these implied that just as most resistant cells depended upon glycolysis or Gln metabolism to counteract the effects of pharmacological stress, oxidative stress, and the nucleotide analog-induced DNA replication blocking [8–11], PDAC heavily relies on lipid accumulation to provide enough energy to sustain the emergence of a resistant phenotype of tumor cells exposed to gemcitabine chemotherapy, which is also verified in lipidomic analysis (Fig. 5) that increased triglyceride accumulation of metabolic adaptations as a critical event in acquiring gemcitabine resistance in PDAC.

Increased lipid production and accumulation in tumor cells are accompanied by enhanced demand for lipid-modifying enzymes [13]. It has been reported that SIK2 increased lipid contents through upregulating the expressions of lipogenic enzymes, resulting in tumor growth in ovarian cancer [33]. In accordance, our results demonstrated that the expression levels of lipogenic enzymes, such as FASN and FADS2, were significantly decreased in gemcitabine-resistant PDAC cells with TGFB2 knockdown when compared with control cells. The family of sterol regulatory element binding factors (SREBFs), which control the expression of a variety of lipogenic enzymes in different cancers, maintains lipid metabolism at the transcriptional level. SREBF1 is a member of the SREBF family, which has been identified in mammalian cells [44]. It can bind to sterol regulatory elements (SREs) in the promoters of target genes and activate genes that are associated with the biosynthesis of triglycerides [45]. Consistently, our results showed that TGFB2 could upregulate the expression levels of SREBF1 at both mRNA and protein levels in gemcitabine-resistant PDAC cells. There is mounting evidence that numerous well-known carcinogenic signaling pathways, including the PI3K/AKT pathway, substantially regulate SREBF1 expression [46]. For example, Thomas Porstmann et al. reported that AKT induced transcription of enzymes involved in lipid biosynthesis via activation of SREBFs [47]. Additionally, PI3K/AKT signaling is a recognized TGF-beta-initiated non-SMAD signaling pathway [48]. Our research further demonstrated that TGFB2 can activate PI3K/AKT signaling to mediate SREBF1-dependent lipogenesis, leading to gemcitabine resistance in PDAC.

It is well-known that altered lipid metabolism is a feature of advanced cancer in various tumors [49]. Enhanced synthesis of lipids may offer more energetic and anabolic demands to compensate for the insufficiency of metabolic demands in resistant tumors, thus sustaining the emergence of resistance [13]. Interestingly, our study demonstrated that compared with parental PDAC cells, gemcitabine-resistant PDAC cells exhibited a marked increase in triglyceride metabolites. Using SREBF1 inhibitors to reduce triglyceride could restore PDAC sensitivity to gemcitabine. Therefore, targeting lipogenic enzymes to reduce triglyceride may be a promising way to develop innovative drugs for gemcitabine-resistant PDAC therapy. In summary, we first demonstrated that N6-methyladenosine-modified TGFB2 triggers lipid metabolism reprogramming to confer pancreatic ductal adenocarcinoma gemcitabine resistance. The combination of TGFB2 inhibitor imperatorin and gemcitabine has a synergistic effect in the gemcitabine-resistant PDAC PDX model. In light of this, our study suggests that TGFB2 and lipid metabolism are promising targets for PDAC clinical management and imperatorin is a potential agent for translation into clinical trials.

## Materials and methods

### Patients and clinical samples

45 patients who were pathologically diagnosed with PDAC and received gemcitabine-based chemotherapy in the First Affiliated Hospital of Sun Yat-sen University were involved in our study. We obtained the paraffin-embedded specimens from the Pathology Department and divided the specimens into two groups according to the Response Evaluation Criteria in Solid Tumors guidelines (RECIST, version1.1), chemo-sensitive patient group (4/45) and chemo-resistant patient group (17/45). 28 PDAC tumors were also collected for the study. The clinical tissue specimens in this study were in accordance with the Declaration of Helsinki and approved by the Ethics Committee of the First Affiliated Hospital of Sun Yat-sen University ([2023]385). Informed consent for the publication of the images from patients was obtained. Informed consent was obtained from all patients.

### Establishment of gemcitabine-resistant cell lines

We established two gemcitabine-resistant cell lines of PDAC cells named BxPC-3 (GR) and CFPAC-1 (GR) by intermittent exposure to gemcitabine. Initially, we added gemcitabine to the complete culture medium to obtain a final concentration of 100 nM. After 24 h of drug exposure, we discarded the medium containing gemcitabine, washed the cells twice with PBS, and replaced it with a fresh complete medium without gemcitabine. We repeated the process of gemcitabine exposure after the cell status returned to normal, as noted through daily observations of cell morphology and growth rate. When the cell morphology and growth rate were no longer affected by drug exposure, the drug concentration was increased to 200 nM, and the process was repeated. When the cells could tolerate a 1000 nM gemcitabine attack, we considered the PDAC cell lines to be resistant to gemcitabine. The intermittent drug exposure process lasted approximately 6 months.

### Establishment of gemcitabine-resistant PDX

To establish a gemcitabine-resistant PDX model, we obtained fresh tumor tissue from patients who were pathologically diagnosed with PADC. The tumor tissues were then cut into 3mm^3^ tissue blocks and immediately implanted into the axillary region of 6-week-old female B-NDG® mice (Biocytogen, Beijing, China). Tumors were removed from the mice when tumor volume reached 1cm^3^ and passaged to the second generation (P2). Similarly, tumors of PDX-P2 were extracted from the mice and propagated through three successive generations in nude mice (P3, P4, and P5). When the tumors reached 4×4×4 mm, the mice were treated with gemcitabine (intraperitoneal injection, 50 mg/kg, twice a week) or PBS during P3-5. It was found that PDX was insensitive to the growth inhibition of gemcitabine after three successive generations of treatment. Then we defined the PDX gemcitabine-generation 5 (GEM-P5) tumor as a gemcitabine gemcitabine-resistant PDX model.

### Cell transfection

Following the methodology described previously, lentivirus packages were used for cell transfection. 293T cells were used for lentivirus packaging, and after 48 h, the supernatant was collected to transfect the targeted cells. Puromycin was used to screen resistant clones for 3–5 days [50].

### Immunohistochemistry

Xenografted tumors from nude mice or tissues from PDAC patients were both preserved in 4% formaldehyde. The specimens were implanted, sliced, and mounted on glass slides by Servicebio (Wuhan, China). According to a previous study [51]. immunohistochemistry (IHC) was used to determine the expression level of TGFB2, Ki67, and PCNA in xenografted tumors from mice and PDAC patients. The DAB staining kit and anti-rabbit IgG HRP-linked antibody (servicebio, Wuhan, China) were used to detect the primary antibodies. The cell nuclei were stained by hematoxylin after DAB staining. The staining results were assessed independently by two experienced observers. The final score was calculated as staining intensity (negative, 0; mild, 1; moderate, 2; severe, 3) multiplying staining area (negative, 0; ≤30%, 1; >30 and ≤60%, 2; >60%, 3).

### Nile red staining

The PDAC-resistant cells were seeded into a six-well plate with a density of 10,000 cells/well. After 48 h’ culture, the cells were washed twice with PBS and fixed in 4% paraformaldehyde for 15 min. Then, we used 5 μg/ml Nile red solution (TOPSCIENCE, Shanghai, China) to stain the fixed cells in the six-well plate followed by PBS washing three times. Finally, the cell nuclei were stained with a DAPI solution (Beyotime, Shanghai, China). The fluorescence from cells was visualized by immunofluorescence microscopy (Leica DMI8).

### Oil red O staining

The cells and tissues were stained with oil red using the Oil Red O (ORO) kit (Leagene Biotechnology Co., Ltd., Beijing, China) as instructed. The red lipid droplets were visualized by microscopy.

### Triglyceride assay

The triglyceride assay was performed using the kits purchased from Applygen Technologies Inc (E1013-105, Beijing, China). In detail, the PDAC-resistant cells were seeded into a six-well plate with a density of 200,000/well. Then, the cells were digested and counted after 2 days of culture. The intracellular level of triglyceride (TG) was analyzed by the kit according to the manufacturer’s instructions. The intracellular level of TG was normalized to the number of cells.

### Animal experiments

To establish a subcutaneous tumor model, the PDAC gemcitabine-resistant cells with different treatments were suspended at a density of 10 million cells/100 µL PBS and injected into the right flank of the 6-week-old female nude mice subcutaneously. After approximately 7 days of tumor growth, the mice were randomized and intraperitoneally injected with PBS or GEM (50 mg/kg, twice a week). For drug combination experiments in vivo, PDX tumors were transplanted into nude mice and the mice were randomized into 4 groups with the dosage and usage of gemcitabine and imperatorin being 50 mg/kg intraperitoneally and 50 mg/kg orally twice a week for 3 weeks, respectively. After three weeks, the mice will be euthanized, and tumor tissue will be extracted for subsequent experiments. The tumor volume was measured every four days by a caliper and calculated as (length × width^2^)/2. All animal experiments were carried out with the approval of the Institutional Review Board of the First Affiliated Hospital, Sun Yat-Sen University ([2023]091). Animal studies were reported in compliance with the ARRIVE guidelines.

### RNA sequencing

The total RNA of PDAC cells (wide type and gemcitabine resistance) was isolated using TRIzol solution. The cDNA library was developed by Novogene (Beijing, China). The paired-end reads were generated by the Illumina Novaseq 6000 and mapped to the human genome hg38 using hisat2. Differentially expressed genes (DEGs) between two groups were identified by the DEGseq R package, with a cut‐off of *p* < 0.05.

### MeRIP-seq and MeRIP-qPCR

Total RNA was extracted from BxPC-3(GR) and CFPAC-1(GR) cells, and Dynabeads mRNA Purification Kit (61006, Invitrogen, USA) was used to further purify the mRNA. RNA fragmentation reagent (AM8740, Invitrogen, USA) was used to fragment RNA before the anti-m6A antibody was utilized for immunoprecipitation. Thereafter, both input and immunoprecipitation RNA samples were processed using NEBNext Ultra RNA Library Prep Kit to prepare sequencing libraries and submitted for sequencing on Illumina HiSeq 2500 by Novogene (Beijing, China) or MeRIP-qPCR analysis.

### Lipidomic analysis

The lipidomics were analyzed in Shanghai Applied Protein Technology Co., Ltd (Shanghai, China). In brief, this project adopts an untargeted lipidomics analysis platform based on the UPLC-Orbitrap mass spectrometry system and combines LipidSearch software (Thermo Scientific™, USA) for integrated analysis including raw data processing, peak extraction, lipid identification, peak alignment, and quantification. The relative content of lipid molecules in samples was obtained on a large scale.

### Statistical analysis

Statistical analyses were carried out using GraphPad Prism 9.0 and Compusyn software. Spearman’s correlation coefficient was used to assess the correlations between the two groups. The data in our study were described as mean ± standard deviation (SD) and compared by *t*‐test or Fisher’s exact test. For all the statistical analyses, *p* < 0.05 were defined as statistically significant.

### Supplementary information

Supplemental information

## Data Availability

All data relevant to the study are included in the article or uploaded as online supplemental information.
