# Supplementary material for: N6-methyladenosine modified TGFB2 triggers lipid metabolism reprogramming to confer pancreatic ductal adenocarcinoma gemcitabine resistance
Source: Oncogene. 2024 Jun 24;43(31):2405–20. doi: 10.1038/s41388-024-03092-3 (PMC11281907; doi:10.1038/s41388-024-03092-3)
Supplement: Supplementary file 1 — Supplemental information [file 41388_2024_3092_MOESM1_ESM.docx]

**Supplemental information**

**N6-methyladenosine modified TGFB2 triggers lipid metabolism reprogramming to confer pancreatic ductal adenocarcinoma gemcitabine resistance**

**Authors:** Ming-Jian Ma^1#^, Yin-Hao Shi^1#^, Zhi-De Liu^1#^, Ying-Qin Zhu^1^, Guang-Yin Zhao^2^, Jing-Yuan Ye^1^, Fu-Xi Li^3^, Xi-Tai Huang^1^, Xi-Yu Wang^1^, Jie-Qin Wang^4^, Qiong-Cong Xu^1*^, Xiao-Yu Yin^1*^.

**Contents**

**Supplemental Materials and Methods**

**Figures S1-S10**

**Tables S1-S2**

**Supplemental Materials and Methods**

**Cell culture and reagents**

PDAC cells BxPC-3 and CFPAC-1 were purchased from Cellcook Co., Ltd. (Guangzhou, China). The cells were cultured in RPMI 1640 medium (Gibco, USA) complemented with 10% fetal bovine serum (Gibco, USA) in a 5% CO2 humidified incubator (Thermo Fisher Scientific, USA) at 37°C.

Gemcitabine, imperatorin, pirfenidone, betulin, fatostain, oleic acid, wortmannin and CMC-Na were purchased from TOPSCIENCE Co. Ltd (Shanghai, China). Recombinant human TGFB2 protein (ab84070) was purchased from Abcam. The TGFB2, METTL14 and IGF2BP2 short hairpin RNA (shRNA) and the primers for RT-qPCR analysis were synthesized from Sangon Biotech Co., Ltd (Shanghai, China). The Supplementary Table 1 and Table 2 lists all the sequences of shRNA and primers. The SREBF1 plasmid were purchased from MiaoLingBio, China. Antibodies for Ki67 (#ab156956) and m6A (#ab151230) were purchased from Abcam. Antibodies for PCNA (#13110), AKT (#4691), p-AKT (#4060), anti-rabbit IgG, HRP365 linked antibody (#7071) and anti-mouse IgG, HRP-linked antibody (#7076) were purchased from Cell Signaling Technology. Antibodies for TGFB2 (19999-1-AP), METTL14 (26158-1-AP), IGF2BP2 (11601-1-AP), PI3K (67071-1-Ig), SREBF1 (66875-1-Ig), and GAPDH (60004-1-Ig) were purchased from proteintech.

**Integrated bioinformatics analysis of PDAC**

RNA-sequencing dataset (GSE140077/148200/154909) and microarray datasets (GSE80617) from the Gene Expression Omnibus (GEO) database were used to screen differentially expressed genes for drug resistance in PDAC. The correlation analysis of TGFB2 and SREBF1 expression in PDAC from GEPIA2 (TCGA) database was conducted. TCGA and Cancer Therapeutics Response Portal (CTRP) databases were applied to further explore TGFB2 implicated mechanism.

**RNA extraction and quantitative real-time PCR**

The total RNA of different cells or groups was extracted by the RNA isolater Total RNA Extraction Reagent (Vazyme, Nanjing, China) as the manufacture’s instruction. RNA reverse transcription was conducted by using HiScript II Q RT SuperMix for qPCR (Vazyme, Nanjing, China) according to the manufacturer’s instructions. The real-time quantitative polymerase chain reaction (RT-qPCR) was performed by the ChamQ Universal SYBR qPCR Master Mix (Vazyme, Nanjing, China) and the relative expression levels were assessed by QuantStudio 6 Flex Real-Time PCR Systems (Applied Biosystems, USA) according to 2−ΔΔCT.

**Western blot**

Total proteins were obtained from cells treated with different treatments by a cell-lysing buffer (BeiyoTime, Shanghai, China) containing inhibitors of proteases (TOPSCIENCE, Shanghai, China) according to manufacturers’ instructions. The proteins were quantified using the BCA Protein Assay Kit (Thermo Scientific, USA) and boiled for 10 min at 95 °C. Precast FuturePAGETM gels (ACE Biotechnology, Nanjing, China) were used to separate the protein samples. The proteins were transferred to polyvinylidene fluoride (PVDF) membranes and then blocked by 5% skim milk in Tris-buffered saline with Tween-20 (TBST) at room temperature for an hour. The indicated primary antibodies were employed to incubate with the membrane at 4°C overnight with gently rocking. After TBST solution washing three times, horseradish peroxidase (HRP)-conjugated secondary antibodies were then incubated with the membrane for one hour at room temperature before being visualized using an enhanced chemiluminescent kit (New Cell & Molecular Biotech, Suzhou, China). GADPH was used as the internal control.

**Cell counting kit-8 assay**

Cell viability was evaluated using the CellTiter-Lumi™ II Luminescent Cell Viability Assay Kit (Beyotime, Shanghai, China). In detail, PDAC resistant cells was seeded into a 96-well plate at a density of 2000 per well. After adhesion, different treatments were applied to PDAC resistant cells, and the baseline fluorescence value was measured in accordance with the manufacturer's instruction. The Fluorescence values were assessed on the 1st, 2nd and 3th day after adhesion. The fluorescence values were positively correlated with the cell counts and could be interpreted as the number of cells per well.

**Apoptosis assay**

BxPC-3(GR) and CFPAC-1(GR) cells from different treatment group were seeded into a six-well plate and cultured for 48 hours before detecting apoptotic rates. In accordance with the manufacturer's instructions, the cells were collected and stained using the Annexin V-APC / 7-AAD cell apoptosis test kit (BB-4101, BestBio, Shanghai, China). The apoptosis rate was estimated by a flow cytometer (BD Bioscience) after staining.

**RNA decay assays**

After treated with 5 μg/ml actinomycin D (Sigma–Aldrich, USA) for 0, 1, 2 and 3 h, the total RNA of BxPC-3(GR) and CFPAC-1(GR) cells was collected and extracted using TRIzol reagent (Life Technologies, USA). RT-qPCR analysis was then used to determine indicated expression level.

**RIP-qPCR**

BxPC-3(GR) and CFPAC-1(GR) cells were harvested in lysis buffer and incubated with the anti‐IGF2BP2 antibody for immunoprecipitation. The RNA from input and immunoprecipitated samples were isolated using the TRIzol reagent and subjected to qPCR analysis.

**Figure S1**

**
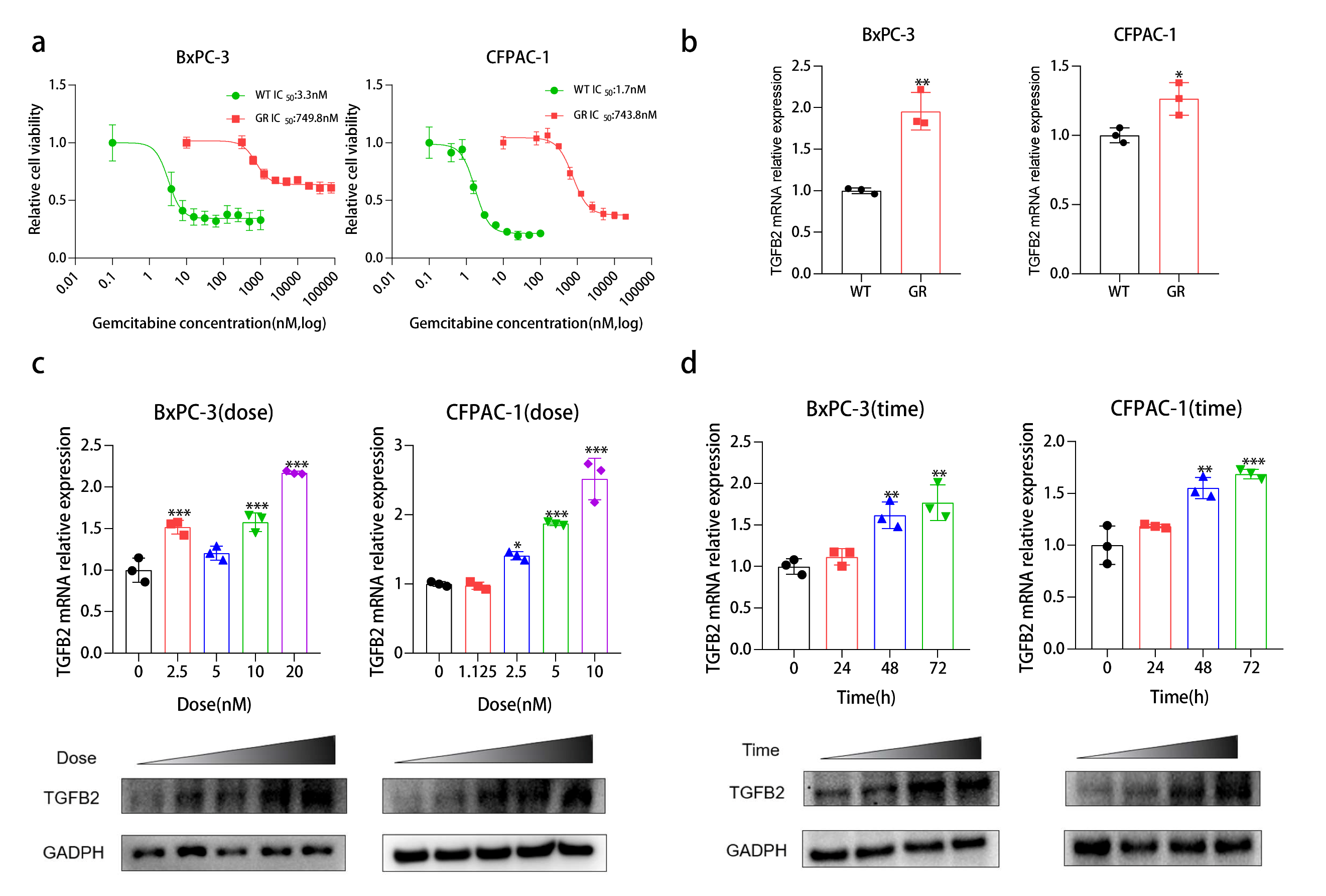
**

**Figure S1. TGFB2 is a crucial gene in gemcitabine resistant PDAC and correlates with poor gemcitabine therapy response.**

**(a)** The IC50 value changes in gemcitabine-resistant BxPC-3/CFPAC-1 cells (GR) and BxPC-3/CFPAC-1 wild type (WT) cells.

**(b)** TGFB2 mRNA level in BxPC-3(WT/GR) cell and CFPAC-1(WT/GR) cell.

**(c)** The TGFB2 level in BxPC-3 and CFPAC-1 cells were measured by RT-qPCR and Western blot at the indicated gemcitabine concentration.

**(d)** The TGFB2 level in BxPC-3 and CFPAC-1 cells were measured by RT-qPCR and Western blot at the indicated gemcitabine time points.

**Figure S2**


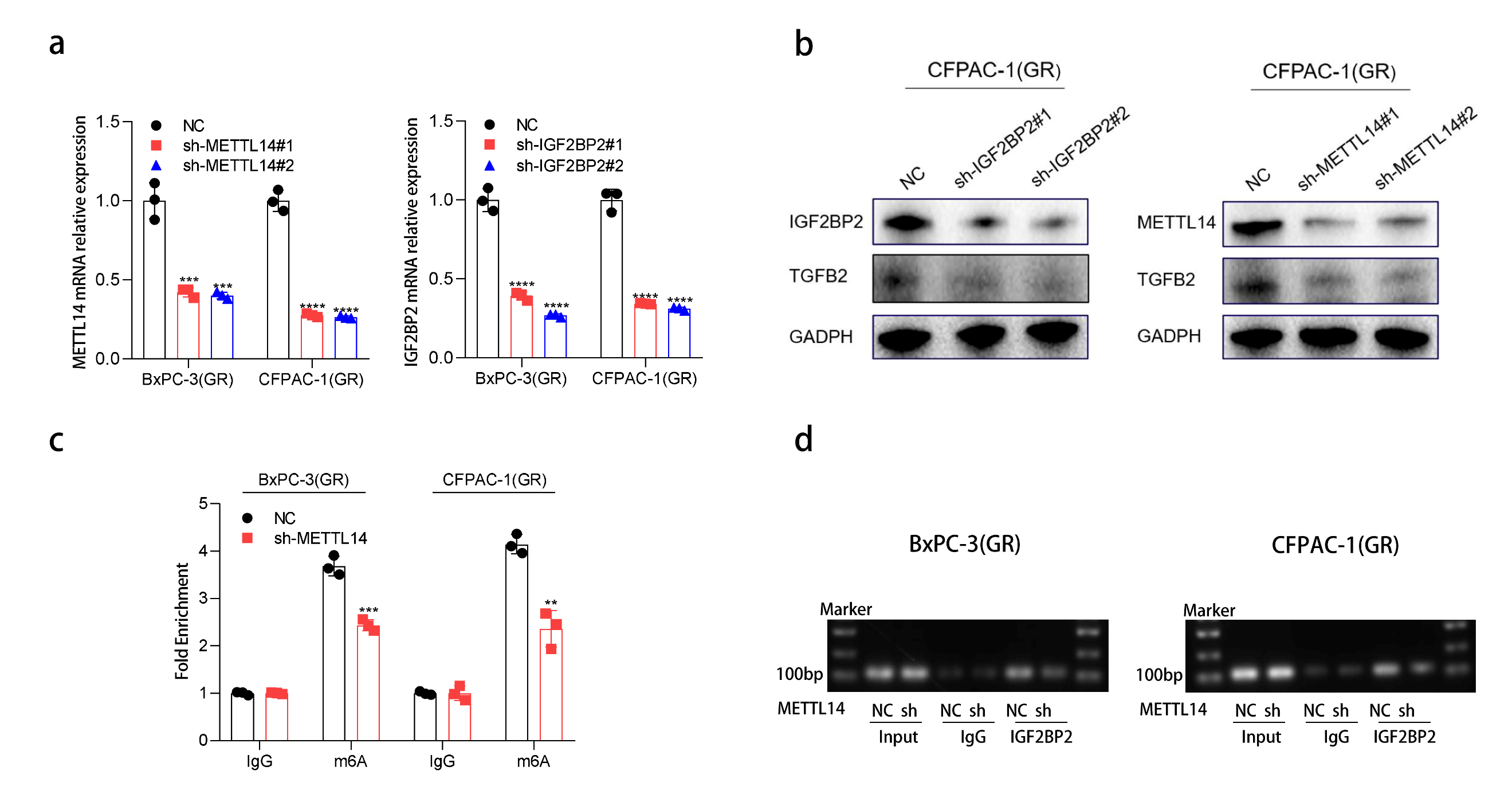


**Figure S2. TGFB2 expression is upregulated by N6‐methyladenosin (m6A) mediated mRNA stabilization.**

**(a)** The mRNA level of METTL14 or IGF2BP2 after METTL14 or IGF2BP2 knockdown in BxPC-3(GR) and CFPAC-1(GR) cells was confirmed by RT-qPCR.

**(b)** The protein level of TGFB2 after METTL14 or IGF2BP2 knockdown in CFPAC-1(GR) cell was confirmed by Western blotting.

**(c)** MeRIP-qPCR analysis of m6A enrichment in the TGFB2 locus in sh-METTL14 or NC BxPC-3(GR) and CFPAC-1(GR) cells. IgG was used as a negative control.

**(d)** Agarose gel electrophoresis using anti-IGF2BP2 antibody showed the affinity of TGFB2 mRNA to IGF2BP2 in sh-METTL14 cells.

**Figure S3**


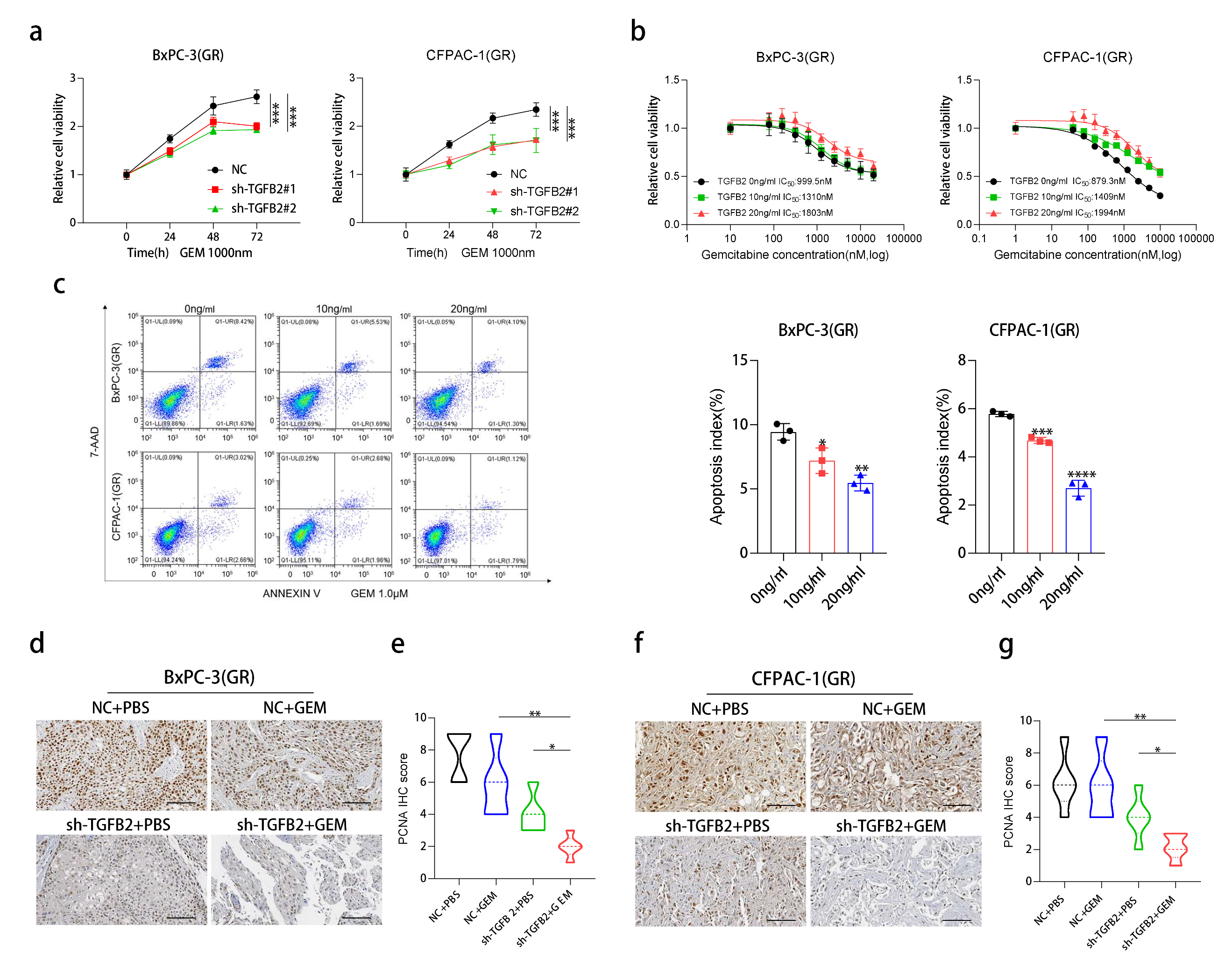


**Figure S3. TGFB2 knockdown potentiates PDAC gemcitabine efficacy in vitro and in vivo.**

**(a)** Cell growth curve of BxPC-3(GR) and CFPAC-1(GR) cells transfected with TGFB2 shRNA or NC treated with 1μM gemcitabine.

**(b)** The IC50 value changes after treated with indicated concentration of recombinant human TGFB2 protein in BxPC-3(GR) and CFPAC-1(GR) cells.

**(c)** Apoptosis assay in BxPC-3(GR) and CFPAC-1(GR) cells treated with indicated concentration of recombinant human TGFB2 protein.

**(d)** Representative IHC staining of PCNA in tumors from each BxPC-3(GR) group (scale bars=100μm).

**(e)** Statistical analysis of IHC staining of PCNA in tumors from each BxPC-3(GR) group.

**(f)** Representative IHC staining of PCNA in tumors from each CFPAC-1(GR) group (scale bars=100μm).

**(g)** Statistical analysis of IHC staining of PCNA in tumors from each CFPAC-1(GR) group.

**Figure S4**


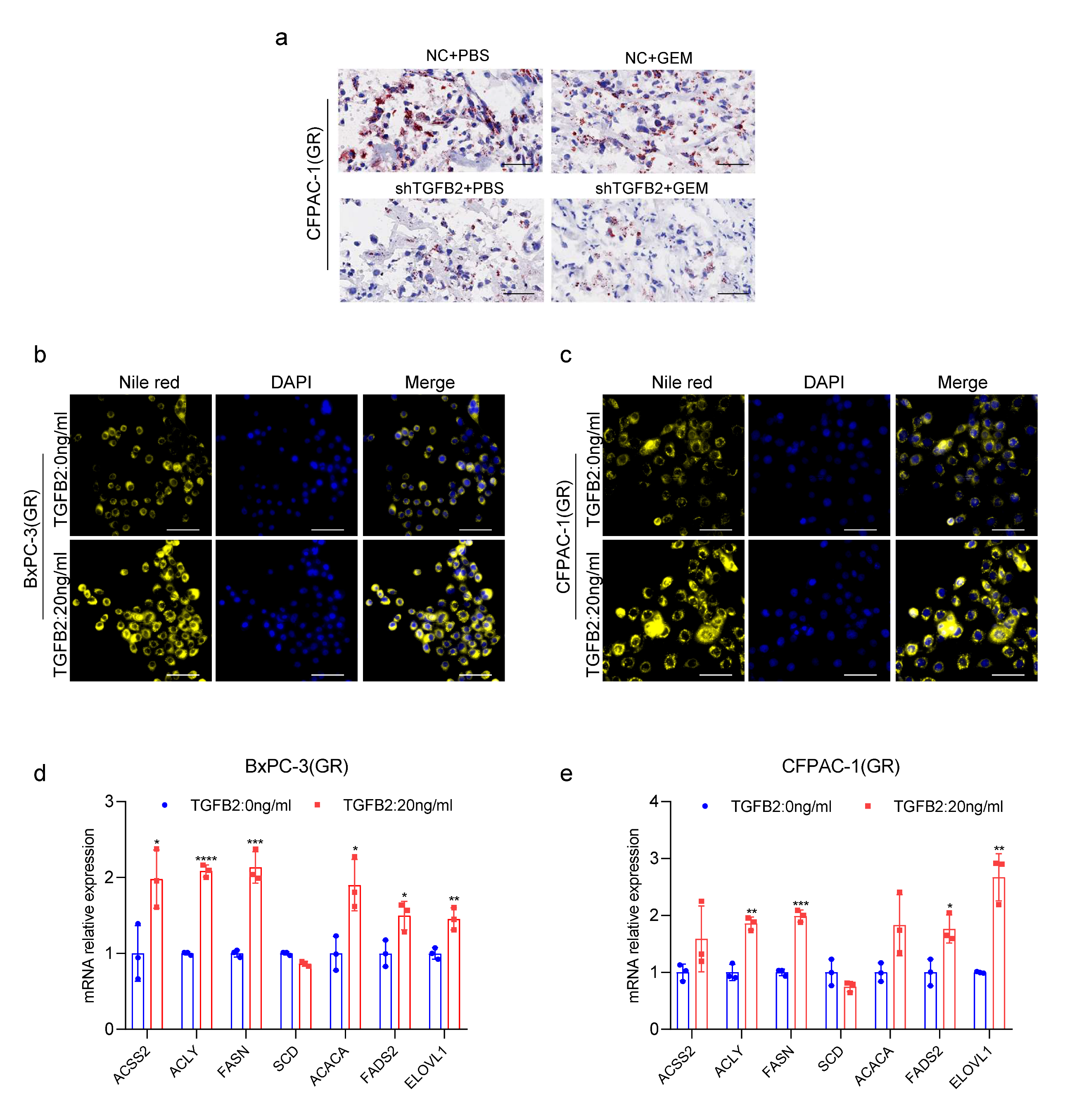


**Figure S4. TGFB2 promotes neutral lipids accumulation by upregulating the expression of lipid synthetase in gemcitabine resistant PDAC.**

**(a)** Tissue neutral lipids were measured in xenograft tumors with CFPAC-1(GR) cells by Oil red O staining (scale bars=50μm).

**(b)** Cellular neutral lipids were measured in BxPC-3(GR) cells treated with indicated concentration of recombinant human TGFB2 protein by Nile red staining (scale bars=100μm).

**(c)** Cellular neutral lipids were measured in CFPAC-1(GR) cells treated with indicated concentration of recombinant human TGFB2 protein by Nile red staining (scale bars=100μm).

**(d)** Expression changes of genes related to lipid synthesis in BxPC-3(GR) cell treated with recombinant human TGFB2 protein.

**(e)** Expression changes of genes related to lipid synthesis in CFPAC-1(GR) cell treated with recombinant human TGFB2 protein.

**Figure S5**


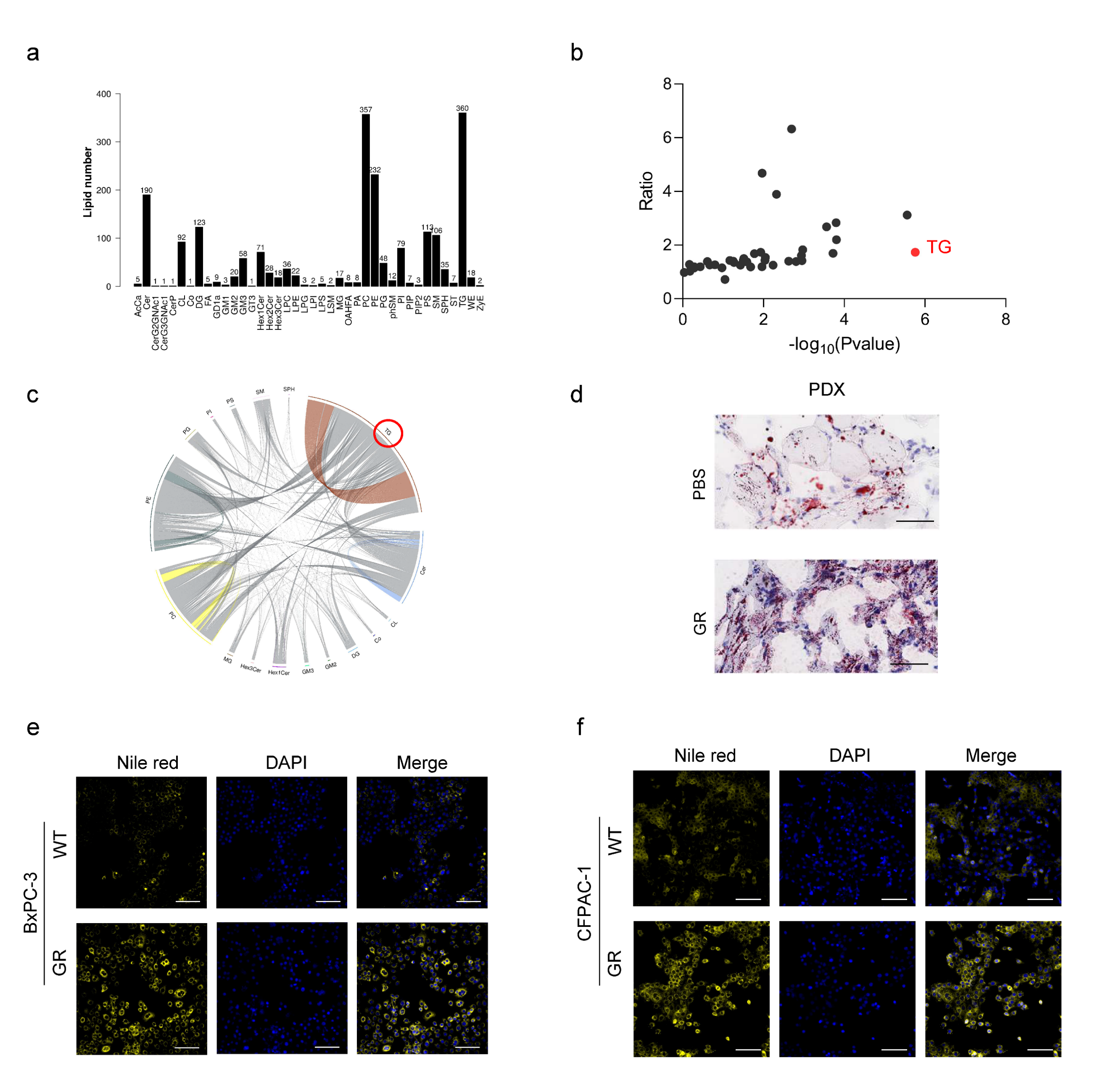


**Figure S5. Increased triglyceride accumulation contributes to promote gemcitabine resistance in PDAC.**

**(a)** 40 classes of lipids were tested in BxPC-3(GR/WT) cells.

**(b)** Scatter plot showed the differential lipid subclasses and molecules of gemcitabine resistance.

**(c)** The chord diagram of lipid-lipid correlation.

**(d)** Tissue neutral lipids were measured in PDX(PBS/GR) by Oil red O staining (scale bars=50μm).

**(e-f)** Cellular neutral lipids were measured in BxPC-3(WT/GR) and CFPAC-1(WT/GR) cells by Nile red staining (scale bars=100μm).

**Figure S6**


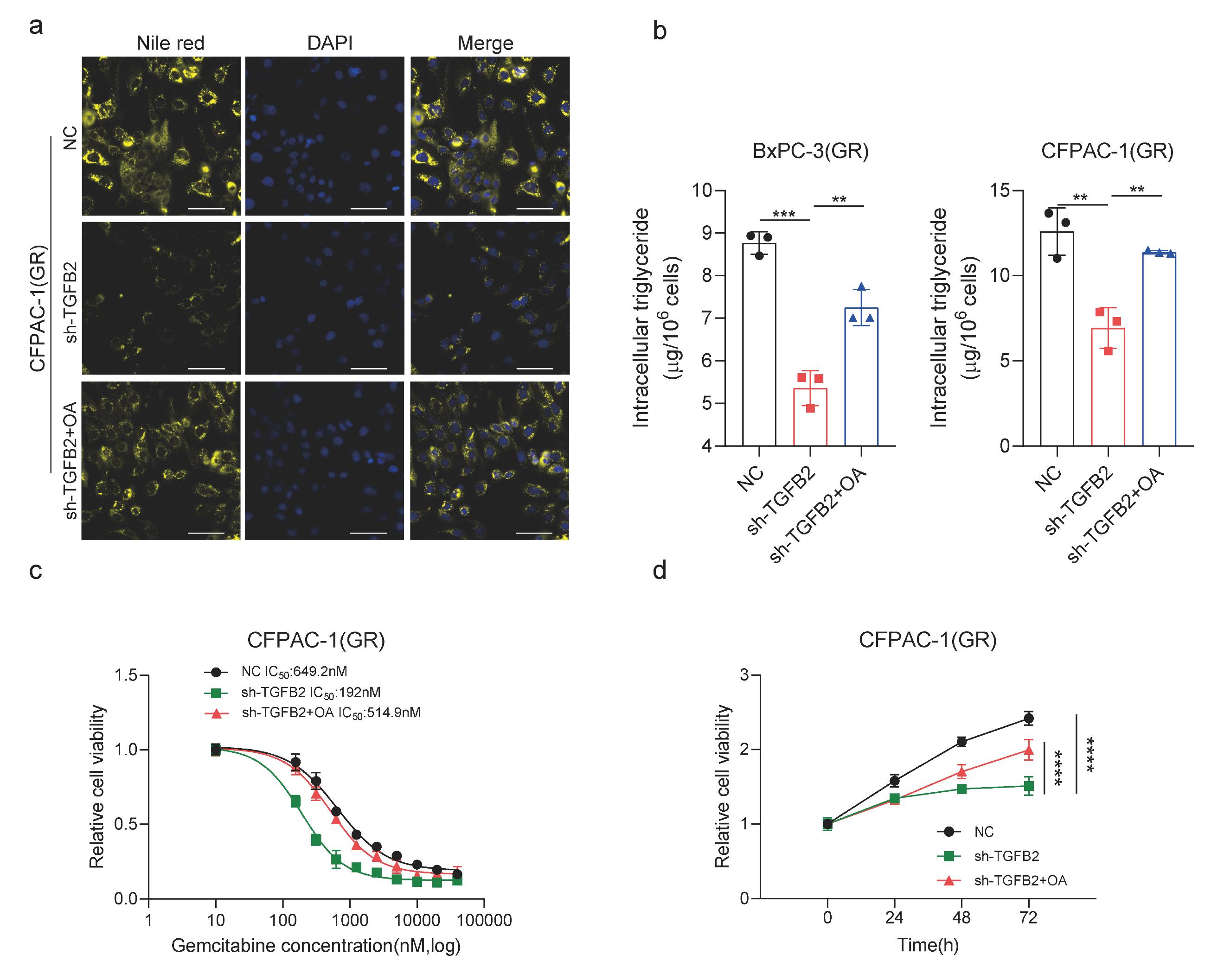


**Figure S6. Oleic acid recovered the drug-resistant phenotype** **of TGFB2-konckdown cells.**

**(a)** Cellular neutral lipids were measured in CFPAC-1(GR) cell with treatment as indicated by Nile red staining (scale bars=100μm).

**(b)** Triglyceride level was measured in BxPC-3(GR) and CFPAC-1(GR) cells with treatment as indicated.

**(c)** The IC50 value changes with treatment as indicated in CFPAC-1(GR) cell.

**(d)** Cell growth curve of CFPAC-1(GR) cell with treatment as indicated.

**Figure S7**


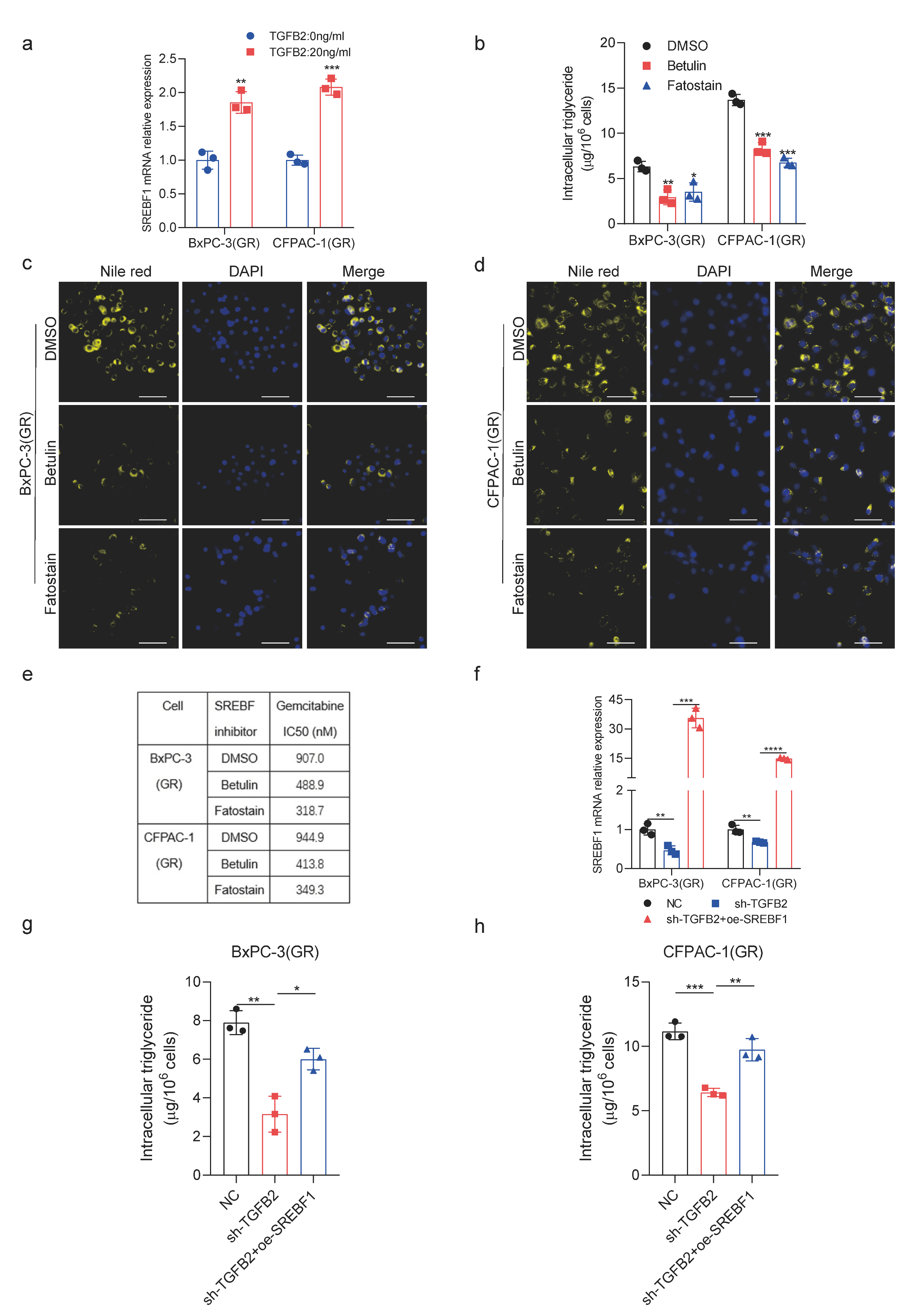


**Figure S7. SREBF1 is critical for TGFB2-AKT-mediated lipid synthesis to promote gemcitabine resistance in PDAC.**

**(a)** The SREBF1 level in BxPC-3(GR) and CFPAC-1(GR)cells treated with recombinant human TGFB2 protein.

**(b)** Triglyceride content was measured in BxPC-3(GR) and CFPAC-1(GR) cells treated with SREBF1 inhibitors.

**(c)** Cellular neutral lipids were measured in BxPC-3(GR) cells treated with SREBF1 inhibitors by Nile red staining (scale bars=100μm).

**(d)** Cellular neutral lipids were measured in CFPAC-1 (GR) cells treated with SREBF1 inhibitors by Nile red staining (scale bars=100μm).

**(e)** The gemcitabine IC50 value changes in BxPC-3(GR) and CFPAC-1(GR) cells treated with SREBF1 inhibitors.

**(f)** Quantitative real-time PCR analyses for expression of SREBF1 in BxPC-3(GR) and CFPAC-1 (GR) cells with treatment as indicated.

**(g-h)** The triglyceride level in TGFB2 knockdown cells with SREBF1 overexpression.

**Figure S8**


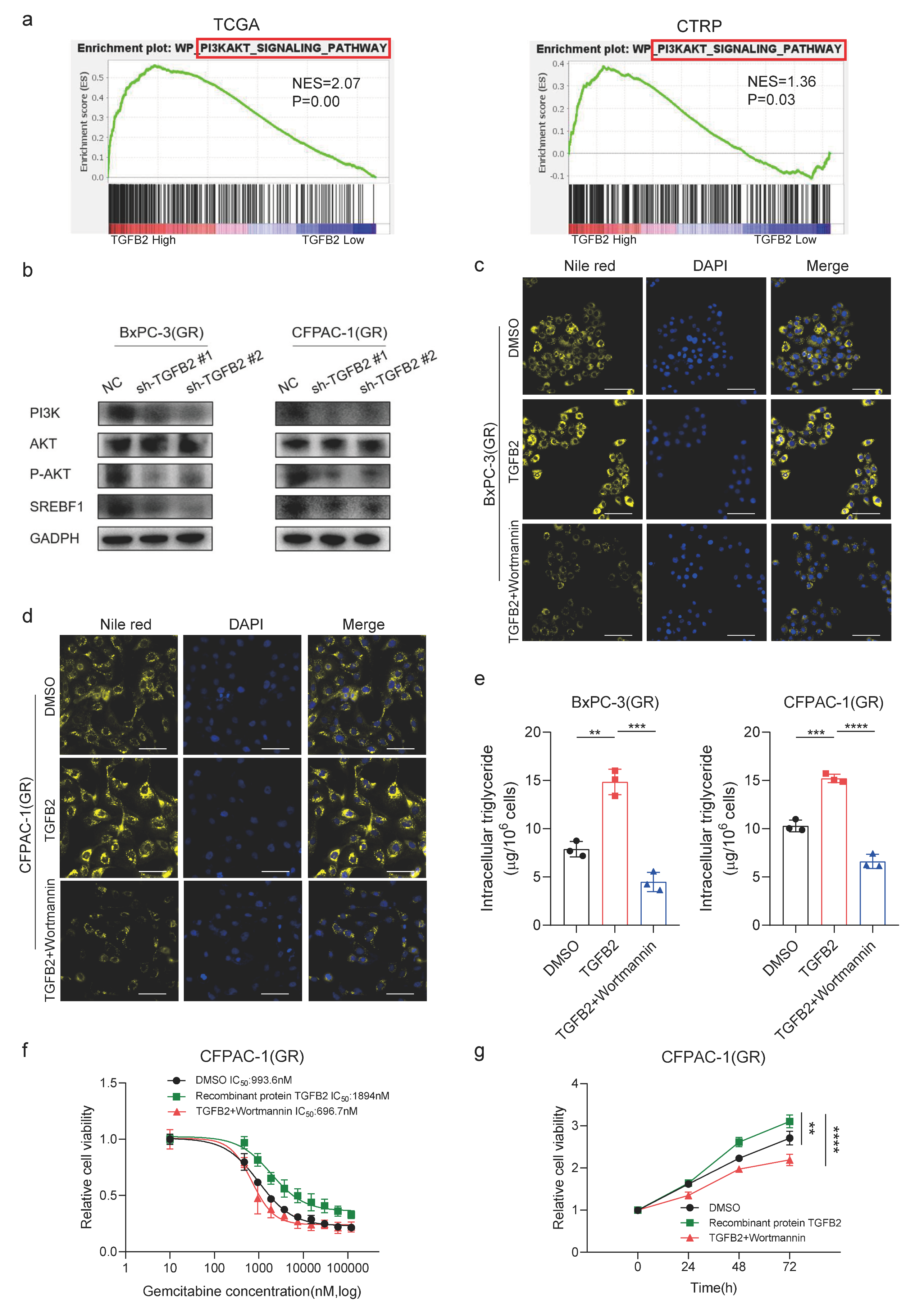


**Figure S8. Wortmannin remarkably suppressed the lipogenesis-promoting effect of TGFB2 overexpression and attenuated the gemcitabine-resistance of PDAC cells.**

**(a)** GSEA enrichment between high and low TGFB2 expression group from TCGA and CTRP database.

**(b)** The PI3K, AKT, phosphorylated AKT (p-AKT) and SREBF1 protein levels after knockdown of TGFB2 in BxPC-3(GR) and CFPAC-1(GR) cells.

**(c-d)** Cellular neutral lipids were measured in BxPC-3(GR) and CFPAC-1(GR) cells with treatment to recombinant protein TGFB2 and wortmannin as indicated by Nile red staining (scale bars=100μm).

**(e)** Triglyceride level was measured in BxPC-3(GR) and CFPAC-1(GR) cells with treatment to recombinant protein TGFB2 and wortmannin as indicated.

**(f)** The IC50 value changes with treatment to recombinant protein TGFB2 and wortmannin as indicated in CFPAC-1(GR) cell.

**(g)** Cell growth curve of CFPAC-1(GR) cell with treatment to recombinant protein TGFB2 and wortmannin as indicated.

**Figure S9**


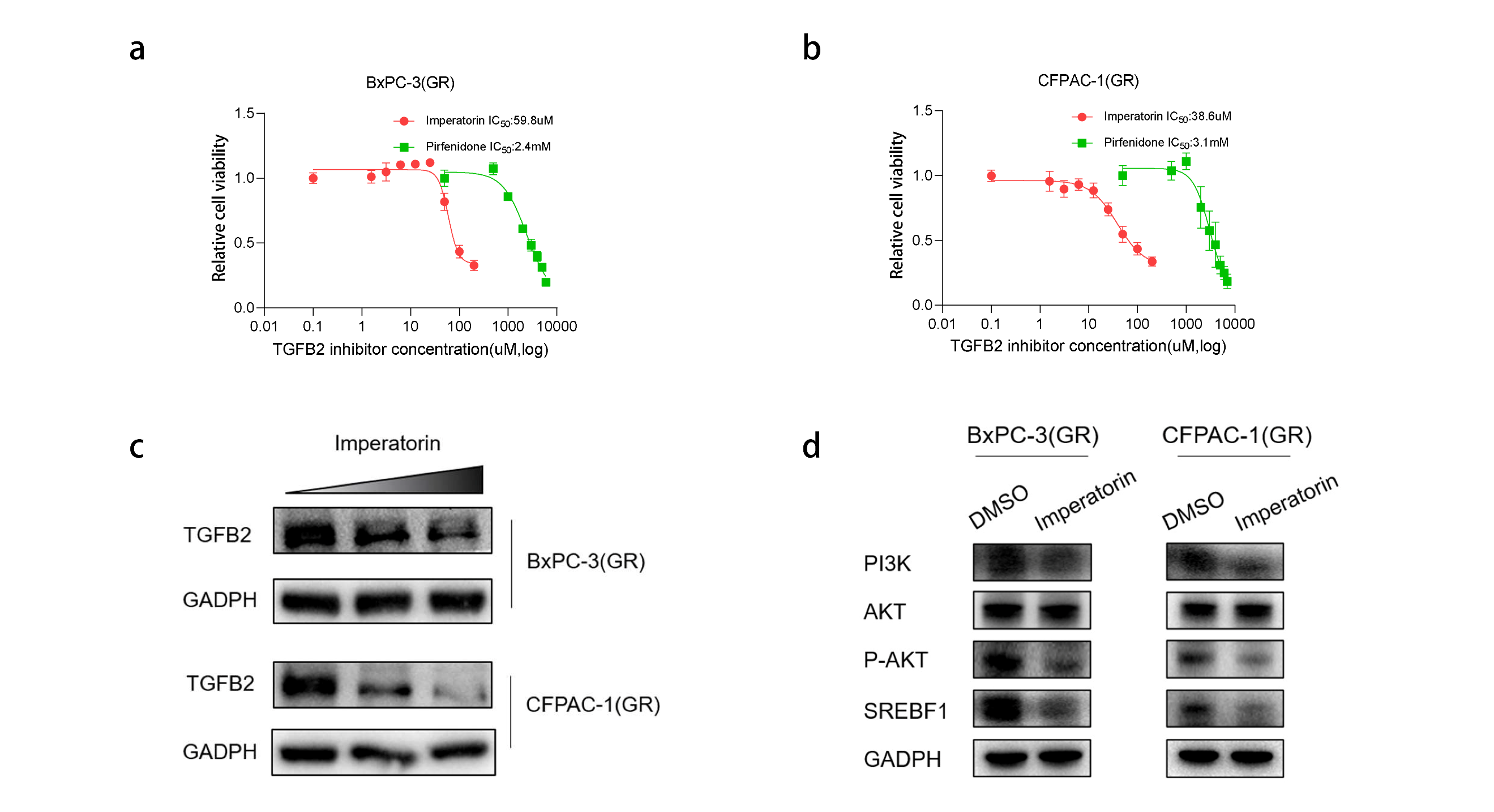


**Figure S9. TGFB2 inhibitor imperatorin exhibits synergistic therapeutic effects with gemcitabine on PDAC.**

**(a-b)** The IC50 value changes after treated with imperatorin or pirfenidone in BxPC-3(GR) and CFPAC-1(GR) cells.

**(c)** TGFB2 level after treated with imperatorin in BxPC-3(GR) and CFPAC-1(GR) cells.

**(d)** The PI3K, AKT, phosphorylated AKT (p-AKT) and SREBF1 protein levels after imperatorin treatment in BxPC-3(GR) and CFPAC-1(GR) cells.

**Figure S10**


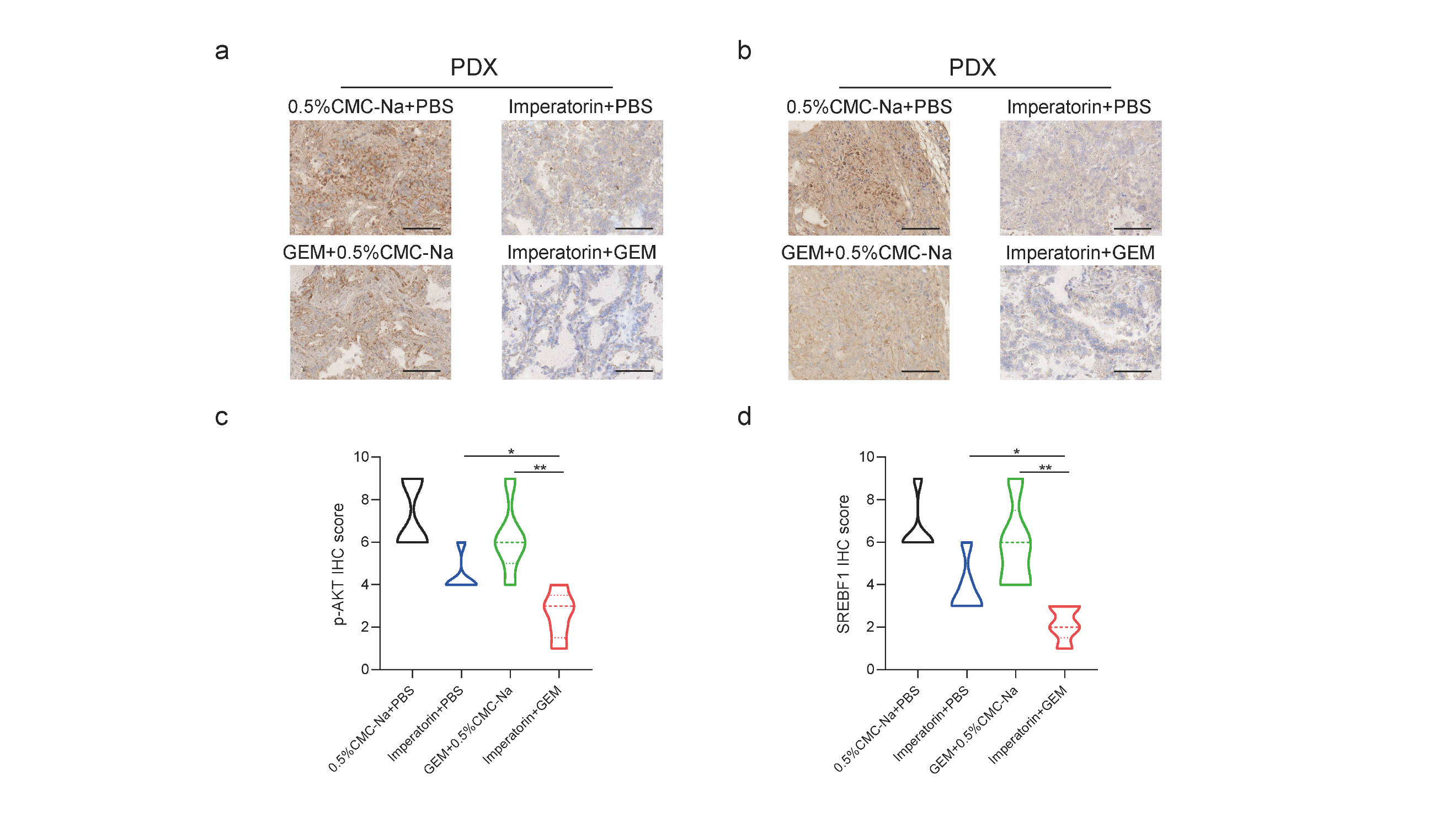


**Figure S10. Imperatorin can significantly enhance the therapeutic efficacy of gemcitabine against chemo resistant PDAC PDX.**

**(a)** Representative IHC staining of p-AKT in tumors with different treatments (scale bars=100μm).

**(b)** Representative IHC staining of SREBF1 in tumors with different treatments (scale bars=100μm).

**(c)** Statistical analysis of IHC staining of p-AKT in tumors from different groups.

**(d)** Statistical analysis of IHC staining of SREBF1 in tumors from different groups.

**Table S1. The sequences of the shRNA used in this study.**

| Names | 5’-3’ Sequence |
| --- | --- |
| shRNA TGFB2-1 | GCGGCCTATTGCTTTAGAAAT |
| shRNA TGFB2-2 | CCAAGATTGAACAGCTTTCTA |
| shRNA METTL14-1 | CCATGTACTTACAAGCCGATA |
| shRNA METTL14-2 | GCTTACAAATAGCAACTACAA |
| shRNA IGF2BP2-1 | CTTAACCAGTGCAGAAGTCAT |
| shRNA IGF2BP2-2 | GCTGTTAACCAACAAGCCAAT |

**Table S2. The sequences of the primers used in this study.**

| Primer |  | 5’-3’ Sequence |
| --- | --- | --- |
| TGFB2 | F | GAGGGATCTAGGGTGGAA |
|  | R | GCTGTGCTGAGTGTCTGAA |
| METTL14 | F | AGTGCCGACAGCATTGGTG |
|  | R | GGAGCAGAGGTATCATAGGAAGC |
| IGF2BP2 | F | AGTGGAATTGCATGGGAAAATCA |
|  | R | CAACGGCGGTTTCTGTGTC |
| ACLY | F | TCGGCCAAGGCAATTTCAGAG |
|  | R | CGAGCATACTTGAACCGATTCT |
| FASN | F | AAGGACCTGTCTAGGTTTGATGC |
|  | R | TGGCTTCATAGGTGACTTCCA |
| SCD | F | TCTAGCTCCTATACCACCACCA |
|  | R | TCGTCTCCAACTTATCTCCTCC |
| ACACA | F | ATGTCTGGCTTGCACCTAGTA |
|  | R | CCCCAAAGCGAGTAACAAATTCT |
| FADS2 | F | TGACCGCAAGGTTTACAACAT |
|  | R | AGGCATCCGTTGCATCTTCTC |
| ELOVL1 | F | TTATTCTCCGAAAGAAAGACGGG |
|  | R | ATGACATGCACGGAAGAGTTTAT |
| ACSS2 | F | AAAGGAGCAACTACCAACATCTG |
|  | R | GCTGAACTGACACACTTGGAC |
| SREBF1 | F | ACAGTGACTTCCCTGGCCTAT |
|  | R | GCATGGACGGGTACATCTTCAA |
| GAPDH | F | GGAGCGAGATCCCTCCAAAAT |
|  | R | GGCTGTTGTCATACTTCTCATGG |
| TGFB2 (meRIP) | F | TGCTATGCAATAGGCACCCT |
|  | R | GGCTCGTTTGAGTTCAAGTTCC |
